# Stock, composition and distribution of the nursing workforce in Brazil: a snapshot*

**DOI:** 10.1590/1518-8345.6937.4287

**Published:** 2024-08-30

**Authors:** Ana Paula Cavalcante de Oliveira, Ana Beatriz Zanardo Mion, Mariana Lopes Galante, Gabriela Di Donato, Carla Aparecida Arena Ventura

**Affiliations:** 1 Universidade de São Paulo, Escola de Enfermagem de Ribeirão Preto, PAHO/WHO Collaborating Centre for Nursing Research Development, Ribeirão Preto, SP, Brazil.

**Keywords:** Health Workforce, Nursing, Organization and Administration, Health Policy, Planning and Management, Brazil, Public Policy

## Abstract

**Objective:**

to analyze the availability (in terms of stock and composition) and accessibility (in terms of geographical distribution) of the nursing workforce in Brazil.

**Method:**

this is a descriptive, cross-sectional study with retrospective data collection, identified by combining databases available on institutional websites and structured according to indicators from the World Health Organization’s “National Health Workforce Accounts”. The study considered nursing professionals at senior level (nurses) and middle level (nursing auxiliaries and technicians). Indicators of stock, composition, distribution (by age group and gender) and the ratio of nurses to doctors were included.

**Results:**

there was an increase in the number of personnel between 2005 and 2010, mainly in middle and technical level professionals. There are more personnel aged between 36 and 55, with a predominance of women in all categories, despite the increase in men. There was an uneven distribution of personnel across the country’s regions, with the Southeast having the largest number of professionals. The ratio of nurses to doctors is less than one in the South and Southeast.

**Conclusion:**

despite the large number of nurses, their distribution is uneven. The growth of nursing technicians has significantly outstripped that of nurses, indicating more intensive technical training policies than those found in higher education.

## Highlights

**(1)** The nursing workforce is still unevenly distributed in Brazil.

**(2)** There is a difference in the composition of the nursing team.

**(3)** The workforce must be planned to guarantee care for the population.

## Introduction

The health workforce (HWF) is a fundamental component of health systems and services. The development of capable, motivated, and supported health workers is essential to the delivery of health care and to overcoming the obstacles that stand in the way of achieving national and global health goals. However, global HWF estimates show a shortage of 15 million workers in 2020, with a projected reduction to 10 million by 2030^1^. In addition to the shortage, many countries face major challenges in their HWF, such as the inadequate mix of skills and inequities in geographical distribution^2^.

Analyzing the current situation of HWF through reliable data and comprehensive research is essential for better understanding the forces driving worker shortages, skill mixes and geographical imbalances, as well as for planning and developing effective policies to address these issues^2^. Thus, the use of evidence for planning the HWF is of paramount importance for building and consolidating resilient health systems and services. This relevance has been recognized and evidenced in the face of the challenges faced worldwide in an international emergency scenario such as the COVID-19 pandemic^3^. In this sense, political will and international cooperation are essential for the development and implementation of targeted national plans aligned with the use of knowledge in building and strengthening health systems. Especially in a context in which policymakers and managers are challenged to ensure the availability and accessibility of healthcare for the entire population, considering the increased demand for healthcare professionals in a context of shortages and poor distribution of these professionals^4^.

The literature emphasizes the contribution of the nursing and midwifery workforce to improvements in health, using a people-centered model of care, favoring proximity to users of health services and the community. Nursing and midwifery, in particular, accounted for more than 50% of the shortage of health workers worldwide^5^ ^)-(^ ^6^.

The creation of the Brazilian National Health System (*Sistema Único de Saúde*, SUS), which established the universal right of access to health care, has led to the expansion of public policies, both in terms of access and population coverage. Despite the expansion of nursing professionals’ workplaces, especially within the SUS and Primary Health Care, attracting and retaining this workforce is a huge challenge for health managers^7^ ^)-(^ ^8^. In this context, Brazilian nursing, which comprised approximately 70% of the country’s HWF in 2019, totaling more than 2.7 million professionals qualified to practice the profession, and representing a density of 12.97 professionals per thousand inhabitants in 2022, has an important role in the health system, far beyond its numerical representation^4^ ^)-(^ ^9^.

Considering that the difficulty in implementing policies or strategies, as well as the lack of political support for monitoring programs and policies, are considered significant limitations that reflect on the decision-making process in the Brazilian context, this study aimed to analyze the availability (in terms of stock and composition) and accessibility (in terms of geographical distribution) of the nursing workforce in Brazil. 

## Method

### Study type

This is a descriptive, cross-sectional study with retrospective data collection, which followed the STROBE guide.

### Study population

Information was collected on the nursing workers who make up the nursing team, i.e. nursing professionals (nurses) and mid-level associate (nursing auxiliaries and nursing technicians). According to the 2008 International Standard Classification of Occupations (ISCO-08): professional nurse (ISCO code 2221) and associate nursing professional (ISCO code 3221) are equivalent to the Brazilian Classification of Occupations (*Classificação Brasileira de Ocupações*, CBO): nurses and equivalents (CBO code 2235) and nursing technicians and auxiliaries (CBO code 3222), according to the Professional Practice Law (Law No. 7,498 of June 25, 1986), which provides for the professional practice of nursing in the country. In relation to medical professionals, ISCO-08 code 2211 and 2212 and CBO code 2002 were used.

### Theoretical model and indicators for analyzing human resources for health

To guide the field of study, the conceptual framework based on the sequence of four critical dimensions: Availability, Accessibility, Acceptability and Quality (AAAQ)^10^ was used, which presents the dimensions of availability, accessibility, quality and acceptability applied to HWF. The concept of availability is presented as the sufficient supply and stock of appropriate health professionals, with the relevant skills, production and combination of skills that correspond to the health needs of the population. 

Accessibility is understood as: the equitable distribution of health professionals in terms of travel and transport time (spatial); opening hours and the presence of the corresponding workforce (temporal); infrastructure attributes (physical, for example, buildings adapted for the disabled); referral mechanisms (organizational) and the direct and indirect cost resources of the services, both formal and informal (financial)^10^.

To structure data collection, the National Health Workforce Accounts (NHWA) tool, developed by the World Health Organization (WHO) in its Portuguese version (2020)^11^ was used. The analysis presented in this article focused on the availability of professionals - in terms of their number (density in relation to the national population) and composition (gender, age, level of training and ratio between nurses and doctors); and spatial accessibility (distribution by region), broken down by category, according to Figure 1.

**Figure 1:** National Health Workforce Accounts indicators and complementary indicators selected for information collection and analysis

Element of the conceptual four AAAQ*	Indicator area	Indicator name	Description used
Availability	Stock of active health workforce	Health professional density.	Numerator: Number of health professionals, defined in number of people. Denominator: Total population.
Availability	Distribution	Distribution of professionals by age group.	Numerator: Number of active health professionals in a specific age group. Denominator: Total number of active health professionals, defined in number of people.
Accessibility	Stock of active health workforce	Professional density at subnational - regional level.	Numerator: Number of active health professionals in subnational administrative units, defined in number of people. Denominator: Total population at sub-national level.
Availability	Complementary	Ratio of nurses professionals to doctors - at national and regional level and by category.	Numerator: Total number of nurses/nurses licensed to practice. Denominator: Total number of doctors licensed to practice^†^.

*The AAAQ framework is a sequence of four critical dimensions to analyze: Availability; Accessibility; Acceptability and Quality; †According to the Organization for Economic Cooperation and Development (OECD)^12^, professionals licensed to practice are those who have training and qualification requirements and a license (registration/qualification) to practice. The concept of active professionals refers to those who have training and qualification as prerequisites for providing services to patients and communities, management, or teaching. Practicing professionals are those who provide direct services to patients and communities

### Data collection and analysis

The data was identified through a combination of databases available on institutional websites: a) the Interagency Health Information Network (RIPSA)^13^ platform, a network made up of governmental and non-governmental institutions^14^: the number of professionals able to work ( auxiliaries, technicians and nurses) by gender and by state in the years 1990, 1995 and 2007 and the number of professionals (auxiliaries, technicians and nurses) registered by state in the years 1990 to 2008 and 2010; b) Federal Nursing Council (Cofen), the body responsible for regulating nursing professions in the country, which aggregates data from the Regional Nursing Councils (Coren) through the Cofen/Coren system: number of professionals able to practice by state, category, age and gender of registered professionals; c) National Health Workforce Accounts (NHWA)^15^, developed by the World Health Organization (WHO), which is based on the conceptual framework of the health labor market^11^: data on gender, age of nursing professionals for the years 2014, 2017, 2019, which may vary between those qualified to practice and those in practice; and d) The Brazilian Institute of Geography and Statistics (*Instituto Brasileiro de Geografia e Estatística*, IBGE)^16^, the country’s main provider of data and information, meeting the needs of various segments of civil society and government bodies^17^: data on the Brazilian population, by federative unit and by region, was obtained from the IBGE’s population estimates published in the Federal Official Gazette.

Although most of the proposed indicators only consider active professionals, the level of activity of the professionals used in the analysis varied according to the information available in the databases. Information available in published reports was also collected, such as: a) Integrated Management Report of the Federal Nursing Council 2019^18^: presents the number and gender of professionals - nurses and nursing auxiliaries and technicians - by state for 2019; b) Business Intelligence Commission Report Product 2^19^: displays data on nursing professionals registered with the Regional Nursing Councils (Coren) in the states by professional category, gender and age group in 2010; c) Business Intelligence Commission Report^20^: with data on nursing professionals registered with the Corens in the states by professional category, gender and age group in 2011, and d) Profile of Nursing in Brazil (Final Report)^21^, resulting from the research project whose central objective was to outline the profile of the nursing team in Brazil. The document identified nursing professionals by age group in 2013.

Data was collected from December 2022 to April 2023 by two researchers simultaneously. Although the authors followed the same data search method for each database mentioned above, there was a lack of information, which made it impossible to collect all the years sequentially in all the databases used. 

The data found was organized in the Excel application and the indicators described in Figure 1 were calculated, taking into account, in addition to the percentage increase (growth rate), the mean number of professionals by gender, by category (nurses, technicians and nursing auxiliaries) and by age.

As far as the Ethics Committee is concerned, despite being a descriptive study, this article is part of a larger study that was submitted to and approved by the Ethics Committee of the Ribeirão Preto School of Nursing (CAAE: 43184621.0.0000.5393 and Opinion Number: 4.859.633).

## Results

### Workforce availability

**Figure 2:**
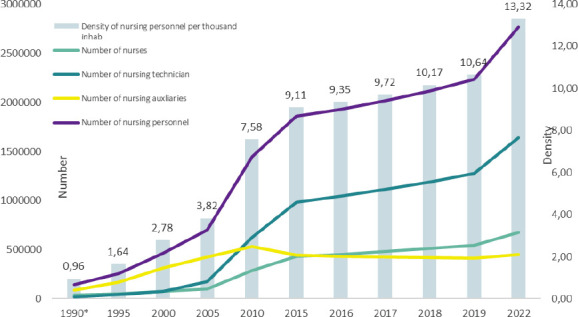
Evolution of the number and density of nursing personnel per thousand inhabitants. Brazil, 1990-2022

Source: Number of registrations of nursing personnel by category: Federal Nursing Council (Cofen)^9^ and Interagency Health Information Network (RIPSA)^13^. Population data obtained from the Brazilian Institute of Geography and Statistics (IBGE)^16^

The total number of professional nursing registrations in the Federal Nursing Council (*Conselho Federal de Enfermagem*, Cofen) and Regional Nursing Council (*Conselho Regional de Enfermagem*, Coren) system was 2,767,741 in December 2022^9^. When analyzing its composition, it was possible to identify that it is mostly made up of mid-level nursing professionals and technicians, 75.47% (totaling 2,088,700 nursing technicians and auxiliaries) and a minority of 24.53% of higher education (679,041 nurses). In relation to the density of nursing professionals per thousand inhabitants in the country, there was a significant increase between 1990 and 2022, from 0.96 to 12.97, respectively. Although this increase is constant and has been observed over the years, it is most significant between 2005 and 2010, when the density of personnel in the country practically doubled in five years, going from 3.82 to 7.58, respectively. Part of the reason for this increase were mid-level professionals, with nursing technicians rising from 0.94 to 3.24 and auxiliaries, from 2.33 to 2.80 in this period. Another moment to observe is the period from 2019 to 2022, which is during the pandemic, when the growth in the density of nursing personnel over three years is almost 22% (from 10.64 to 12.97), whereas in the previous period, the growth was approximately 14% (from 9.35 to 10.64), as illustrated in Figure 2.

**Figure 3:**
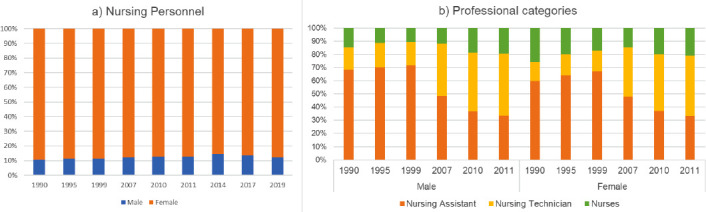
Characterization of the nursing workforce by gender, total and by category. Brazil, 1990-2019

Source: Number of professional nursing records, 2011: Federal Nursing Council, Cofen^20^; 1990 to 2010: Interagency Health Information Network (RIPSA)^13^; 2014 to 2019: National Health Workforce Accounts (NHWA)^15^; Number of nursing professional registrations by category and gender: Federal Nursing Council (Cofen)^19^ ^)-(^ ^20^ and Interagency Health Information Network (RIPSA)^13^

As for the characterization of professionals, when analyzing the distribution of nursing personnel (without distinguishing between categories) by gender, it can be seen that, despite a slight increase in the number of male professionals, women continued to predominate in the last two decades, from 1990 to 2019, representing 88.00% in 2019, as shown in Figure 3, sub-item (a). A more detailed analysis of the professional categories from 1990 to 2011 reveals a slightly more pronounced increase in the male contingent among higher education professionals, from 6.42% in 1990 to 11.86% in 2011, as shown in Figure 3, sub-item (b).

From 2010 to 2019, when we analyzed the age distribution of nursing personnel, we found that, despite the data coming from different sources, there was a predominance of individuals between 36 and 55 years old. However, in 2017 and 2019, there was a decrease in the percentage of nursing personnel under 35. The data showed that personnel under the age of 35 accounted for more than 35% in all the years studied, ranging from 35.70% to 45.90%, while those aged 56 or over accounted for more than 6% of all nursing personnel, with percentages ranging from 6.09% to 9.81%. This information was obtained from the number of records of nursing personnel by category for the years 2010 and 2011^9^, for 2013^21^ and, for 2014, 2017 and 2019, extracted from the NHWA^15^.

To analyze the skills combination, we used the density and ratio of nursing and medical professionals in the country, Tables 1 and 2. When we compared the density of nurses to that of doctors per 10,000 inhabitants for the year 2022, it was slightly higher for nurses (31.83 and 27.38 respectively), reaching a national ratio of 1.16 nurses professionals per doctor in the same year. The biggest difference between the densities of these professionals can be seen in the North and Northeast regions, which also had the highest ratios of nurses to doctors in the country (1.98 and 1.64 respectively). At the other extreme are the South and Southeast, which have the lowest nurse to doctor ratios (0.95 and 0.99 respectively). When analyzing the ratio of nursing professionals to doctors, a national ratio of 4.74 was identified, ranging from 3.83 nursing professionals per doctor in the South to 8.52 in the North.

**Table 1:** Density of nursing personnel, total and by category, per 10,000 inhabitants and ratio of nursing professionals, total and nurses, per doctor. Brazil, 2022

Region	Density per 10,000 inhabitants							Ratio	
	ENF^†^	Téc Enf^‡^	Aux Enf^§^	Prof Enf^ǁ^	Doctors*	ENF^†^ and doctors	Prof Enf^ǁ^ and doctors	ENF^†^ by doctor	Prof Enf^ǁ^ by doctor
North	28.72	85.65	9.29	123.66	14.52	43.21	138.18	1.98	8.52
North East	31.64	72.40	11.71	115.75	19.29	50.92	135.04	1.64	6.00
Southeast	33.56	78.67	34.87	147.10	33.90	67.46	181.00	0.99	4.34
South	26.80	73.22	13.07	113.09	29.52	56.32	142.61	0.95	3.83
Midwest	35.91	78.39	7.89	122.19	31.02	66.93	143.21	1.16	3.94
Total	31.83	76.79	21.12	129.75	27.38	59.22	157.13	1.16	4.74

Source: Number of registrations of nursing professionals by category: Federal Nursing Council (Cofen)^9^; number of inhabitants (Brazilian population) in 2022: Brazilian Institute of Geography and Statistics (IBGE)^16^ and number of registrations of medical professionals: Medical Demography in Brazil 2023^22^

*The number of medical professional records was used to enable comparison with the nursing data; †ENF = Nurses; ‡Téc Enf = Nursing technicians; §Aux Enf = Nursing auxiliaries; ǁProf Enf = Nursing professionals

**Table 2:** Number of nursing personnel, total and by category, and growth rate by region. Brazil, 2000 and 2022

Region	ENF*			Téc Enf†			Aux Enf‡			Prof Enf§		
	2000	2022	Rate	2000	2022	Rate	2000	2022	Rate	2000	2022	Rate
North	4.150	54.305	1209%	3.408	161.936	4652%	13.683	17.559	28%	21.241	233.800	1001%
North East	17.959	182.442	916%	10.912	417.526	3726%	52.550	67.544	29%	81.421	667.512	720%
Southeast	38.054	300.804	690%	44.627	705.131	1480%	185.621	312.521	68%	268.302	1.318.456	391%
South	9.263	81.487	780%	8.822	222.594	2423%	42.059	39.746	-5%	60.144	343.827	472%
Midwest	4.695	60.003	1178%	10.074	130.965	1200%	16.254	13.178	-19%	31.023	204.146	558%
Total	74.121	679.041	816%	77.843	1.638.152	2004%	310.167	450.548	45%	462.131	2.767.741	499%

Source: Numbers of registered nursing professionals by category for the year 2022, obtained from the Federal Nursing Council (Cofen)^9^ and for the year 2000, obtained from the Interagency Health Information Network (RIPSA)^13^

*ENF = Nurses; †Téc Enf = Nursing technicians; ‡Aux Enf = Nursing auxiliaries; §Prof Enf = Nursing professionals

When analyzing the availability of this group of professionals in the country, as shown in Table 1, there was a national density of 59.22 nurses and doctors, as well as 57.13 nursing professionals and doctors per 10,000 inhabitants. In addition, it was possible to identify the lowest density in the country in the North and Northeast regions, both in terms of professionals with higher education (43.21 and 50.92 nurses and doctors per 10,000 inhabitants, respectively), and in terms of mid-level/technical professionals (138.18 and 135.04 nursing professionals and doctors per 10,000 inhabitants).

### Workforce accessibility

When analyzing the distribution of nursing personnel among the country’s regions, it can be seen that the largest number is concentrated in the Southeast, followed by the Northeast, with the same distribution in the different professional categories. The North is the region with the lowest number of nurses in the country, but when analyzing the number of nursing professionals, nursing auxiliaries and nursing technicians, the region with the lowest representation is the Midwest, as shown in Table 2.

Although the Southeast is the most representative region in the country in terms of the number of nursing personnel in its different categories, it showed the lowest growth during the period from 2000 to 2022, considering the total number of nursing professionals and nurses. The North was the region with the greatest growth in relation to the different categories analyzed, except for nursing auxiliaries. However, when looking only at nursing auxiliaries, the South and Midwest regions showed a decrease in the category, as shown in Table 2. 

It is therefore important to highlight the density of professionals, i.e. the number of professionals per inhabitant in a given location. In relation to this indicator, the Southeast region, despite having the lowest growth rate in the period analyzed by this study, is the region with the highest density of nursing professionals in the country (and number of professionals), since it has the second highest density of nurses and the highest density of mid-level and technical professionals, with a special participation of nursing auxiliaries (34.87 in contrast to the scenario of deceleration of this category). When looking at the density of nurses, the Central-West region is first, even though it has the fourth lowest number of nurses of all the regions. 

Although the Northeast region stands out in terms of the number of nursing personnel, ranking second in all categories, it has the second lowest density of nursing professionals and the lowest density of technicians and assistants combined. The great growth of the Northern region is not yet reflected in better densities of professionals, representing the second lowest density in the country. The South has the lowest density of nursing professionals and nurses in the country and the second highest density of nursing auxiliaries, as can be seen in Table 1.

## Discussion

The density of nursing in the country, of approximately 130 nursing professionals and 32 nurses per 10,000 inhabitants in 2022, has been identified as higher than the mean for countries in the Latin American and Caribbean Region (LAC) and the Organization for Economic Cooperation and Development (OECD), which have just over 35 and 103 nursing professionals per 10,000 inhabitants respectively for the same year (information available for LAC for 33 countries, and OECD for 38 countries)^23^, demonstrating an apparent “excess of professionals” in Brazil. It is important to note that the global data may be considering practicing and/or active professionals, while the data analyzed for Brazil refers to professionals licensed to practice, considering the number of registrations with the Cofen/Coren System. 

In addition, in Brazil, in 2022, seven out of ten nursing personnel will have a secondary and/or technical degree (75%), which is the opposite of the global scenario, in which 69% of professionals have a higher education degree (considering data from previous years). In the European region, nurses make up 70% of the nursing workforce (for example, the United Kingdom, with 80% of professionals with higher education)^24^. With the exception of a few countries, such as Canada and the United States (75.46% and 85.74% of professionals with higher education, respectively)^15^ ^),(^ ^24^, the nursing workforce in the Americas is mostly made up of mid-level and/or technical professionals, although at a lower rate than in Brazil (59%)^25^.

During the period analyzed (1990 to 2022), two moments of significant increase in the number of nursing personnel in Brazil were identified. The first occurred between 2005 and 2010, when there was a significant increase in the density of personnel, almost doubling from 3.82 to 7.58. Nursing technicians played a key role in this increase, rising from 0.94 to 3.24. This expansion was driven by national strategies, such as the Nursing Workers Professionalization Project (*Projeto de Profissionalização dos Trabalhadores da Área da Enfermagem*, PROFAE)^26^. The second period of growth in the density of nursing professionals, of around 22% between 2019 and 2022, partially contradicts the results of an international integrative review that examined 43 studies (pre- and post-COVID-19) and indicated a significant increase in the rate of intention to leave the profession after the COVID-19 pandemic, as also observed by the International Council of Nurses (ICN). In addition, a survey of National Nursing Associations showed an increase in the number of nurses leaving the profession. In this context, factors related to this intention and/or abandonment are emphasized: heavy workload, insufficient resources, physical exhaustion and stress^27^ ^)-(^ ^28^. Therefore, it is imperative to examine this scenario in the country, analyzing not only the number of professionals involved in direct care for patients and the community, but also the number of individuals who have chosen to leave this noble profession.

Despite being a young profession, with more than a third of professionals at the start of their careers and less than 9% close to retirement (2019), there is an aging of the profession in the country (2010 to 2019). A younger nursing workforce can help ensure the sustainability of the nursing profession. As older nurses retire, it is important to have a new generation to replace them and continue providing high-quality care^29^. Although this is far from the scenario in some countries in the Americas with substantially older populations, such as Panama and the Dominican Republic (with more than 26% of professionals aged 55 or over)^15^, and countries in Europe, such as Bulgaria, Iceland and the Republic of Moldova (with approximately 40% of professionals aged 55 or over)^30^, this information suggests the need to monitor this indicator and carry out analyses between the different categories, in order to better understand the flows in and out and the needs of professionals.

In relation to the characteristics of the nursing workforce, there is a predominantly female profile. In this study, it was possible to identify an increase in the male contingent, representing 12% of professionals qualified to practice in 2019, a slightly higher increase in the category of nurses (from 6% to 12% 1990-2011). Data on nursing professionals in practice indicate a percentage of 13.7% in 2021 for the country^16^. These figures are close to those identified in other countries in the Americas region, with 13% in 2019^16^, with Argentina standing out with approximately 20% of male nursing professionals in 2020^15^ ^),(^ ^25^.

Although female health workers are responsible for providing the majority of care at all levels and in all settings, as they represent around 67% of salaried workers in the health sector, they encounter barriers at work. These barriers are not faced by male professionals, such as prejudice, discrimination, sexual harassment and violence, in addition to the disproportionate risks that have arisen during the COVID-19 pandemic^31^. The integration of gender and age into HWF strategies is necessary to ensure that sensitive, evidence-based approaches are adopted for planning and managing professionals. The lack of accurate data on gender and age composition in several countries hinders the improvement of working conditions and career structures to support women’s participation and progress in HWF, as well as valuing the contributions of women and their professionals in healthcare systems^32^. 

By estimating the combination of HWF skills by comparing the ratio of professionals, information is obtained on the possibility of efficiency gains and also the ratio of nurses to doctors as a potential indicator of the quality of user care^2^ ^),(^ ^33^. Brazil had a national ratio of 1.16 nurses in 2022 and two of its regions, the South and Southeast, had nurse to doctor ratios below 1 (0.95 and 0.99 respectively). These were identified as lower than the mean for LAC and OECD countries, which correspond to 1.9 and 2.7 nurses for each doctor, respectively for the same year (information available for LAC 32)^23^. Several countries in the region have ratios above this figure, including Dominica, the highest in the region, with 5.5 nurses per doctor. On the other hand, Colombia’s ratio of 0.6 nurses per doctor stands out, at the lowest end of the LAC region.

This study identified a national nurse to doctor ratio of 4.74 in 2022. Considering the OECD and LAC mean, this ratio is relatively higher in Brazil. Furthermore, it is important to reiterate that the nursing workforce has different characteristics (mostly made up of mid-level and technical professionals). It should also be noted that the OECD document presents data on active professionals in Brazil in 2019 (3.4 nursing professionals) and identifies a figure higher than that of these groups of countries (the OECD mean is 2.6 and LAC, 1.6)^34^. It is therefore essential to rethink the combination of health professional skills in the context of an ageing population and an increase in the burden of chronic diseases in the country.

Considering workforce accessibility, density estimates indicate the per capita availability of health workers. The variation in density across geographic regions provides insight into the equitable distribution of healthcare personnel between regions^2^. The background document for the WHO’s Global Strategy on Human Resources for Health: Workforce 2030 estimated the “minimum threshold” of 44.50 doctors, nurses and midwives per 10,000 inhabitants as necessary to offer universal health coverage to the population^35^ ^)-(^ ^36^. When analyzing the availability of this group of professionals in the country, this study found a national density of 59.22 nurses and doctors per 10,000 inhabitants, including mid-level and technical professionals of 157.13 nursing and medical professionals, i.e. both national densities reach this “minimum limit”. When considering only professionals with higher education, the Northern region had a lower density than this established limit (43.21 nurses and doctors per 10,000 inhabitants). However, although this threshold is a valuable metric for international comparisons and monitoring, it is not advisable to use it directly to set national targets. Such an approach disregards the country’s peculiarities, such as health conditions, structures, and the presence of occupational groups other than the three professions in the country. Therefore, it is essential to develop targets adapted to the national context, considering the political priorities for the availability and composition of the Health Workforce (HWF)^2^.

In the last two decades, there has been a movement to expand nursing education in Brazil, with the creation of schools in all regions. This movement has resulted from the democratization of access to higher education and has resulted in more nurses being available on the market^37^. Despite this expansion, nursing schools are concentrated in the states with the highest population density and concentration of income in the country, in line with the distribution of the Gross Domestic Product (GDP) and the economic and social inequalities of Brazil’s regions^37^.

The data identified in this study, for the year 2022, indicates that only the Southeast region had almost 50% of the number of nursing professionals in the country. In this sense, this region also had higher numbers of professionals in previous years, based on a study that analyzed information for nurses in different years of the 2010s^38^. Remote areas can face a shortage of qualified professionals, prolonging regional disparities. In contrast, central and wealthy areas may have a smaller supply of qualified professionals and lower employability^39^.

A study sought to estimate the number of employed nurses that should be redistributed in order to achieve a more adequate geographical distribution of these professionals in Brazil, and identified that the Southeast region should redistribute 8.8% of its nurses among the Brazilian regions, representing around 6,000 nursing jobs to be redistributed in order to achieve a situation of equality in relation to this ratio^40^. In addition, an analysis of the population coverage of nurses in the states of the North and Northeast showed population coverage below 5 nurses per 10,000 inhabitants, with values above 10 nurses per 10,000 inhabitants^38^. 

The study’s limitations refer to the characteristics of each database consulted, given the fragmentation of information, the use of different methodologies in data collection and the level of professional activity. There is a need for standardization in the collection and dissemination of data on health professionals. 

Continuous monitoring and evaluation of the nursing workforce is essential. In this sense, considering the distortions concerning the unequal distribution of the contingent of nursing professionals in the Brazilian states, it is important that the public authorities are responsible for this process, offering nursing courses that are suited to local realities and global training requirements, in the territories that lack nurses, especially in the interior of Brazil^41^.

In short, this study plays a significant role in advancing scientific knowledge by providing a detailed analysis of the availability and accessibility of the nursing workforce in Brazil. By jointly employing descriptive and cross-sectional methods and carefully selected indicators, the researchers were able to draw a comprehensive picture of the situation. The results highlight changes over time, revealing a notable increase in the number of nursing professionals, especially at middle and technical level, between 2005 and 2010. In addition, the unequal distribution of professionals in different regions of the country was identified, with the Southeast region standing out in terms of quantity. The analysis also pointed to persistent challenges, such as the predominance of females in all categories and the need for more robust public policies to diversify and redistribute professionals across Brazil. These findings not only inform future decisions in the areas of health and education, but also offer valuable insights for formulating strategies aimed at improving the quality of health care and promoting equity in access to health care services across the country.

In this sense, the importance of public policies that take statistics into account when planning and implementing policies and strategies is emphasized, in order to reconfigure the distribution of professionals to promote a more equitable allocation in all regions of the Brazilian territory. Of particular note is the effort made with the Program for Valuing Primary Care Professionals (*Programa de Valorização dos Profissionais da Atenção Básica*, PROVAB), which aimed to provide Primary Care and Family Health Strategy teams with nurses, dentists and doctors in remote and more vulnerable areas. 

Finally, there is a pressing need for new research and policies that thoroughly examine the effectiveness of integrating professionals into their roles and workplaces. This is crucial to understanding the growing demand for nursing professionals, a need that seems to intensify every year, despite the increase in the number of these professionals.

## Conclusion

Despite the large number of nursing personnel, there is a disparity in their distribution throughout the country. It is also possible to note that nurses, in addition to being in smaller numbers, have grown much less than nursing technicians in some regions of the country, so that the growth of nursing technicians has outstripped that of nurses, indicating more intensive technical training policies than in higher education.
