# Supplementary material for: Stock, composition and distribution of the nursing workforce in Brazil: a snapshot
Source: Rev Lat Am Enfermagem. 2024 Aug 30;32:e4287. doi: 10.1590/1518-8345.6937.4287 (PMC11368065; doi:10.1590/1518-8345.6937.4287)
Supplement: Translation

# Translation: Estoque, composição e distribuição da força de trabalho de enfermagem no Brasil: uma fotografia*

**DOI:** 10.1590/1518-8345.6937.4288

**Authors:** Ana Paula Cavalcante de Oliveira, Ana Beatriz Zanardo Mion, Mariana Lopes Galante, Gabriela Di Donato, Carla Aparecida Arena Ventura

**Affiliations:** 1 Universidade de São Paulo, Escola de Enfermagem de Ribeirão Preto, Centro Colaborador da OPAS/OMS para o Desenvolvimento da Pesquisa em Enfermagem, Ribeirão Preto, SP, Brasil.

**Keywords:** Mão de Obra em Saúde, Enfermagem, Organização e Administração, Políticas, Planejamento e Administração em Saúde, Brasil, Política Pública

## Abstract

**Objetivo:**

analisar a disponibilidade (estoque e composição) e acessibilidade (distribuição geográfica) da força de trabalho de enfermagem no Brasil.

**Método:**

estudo descritivo e transversal, com coleta retrospectiva de dados, identificados por meio da combinação de bancos de dados disponíveis em *sites* institucionais e estruturados de acordo com indicadores das “Contas Nacionais da Força de Trabalho em Saúde” da Organização Mundial da Saúde. Consideraram-se, para o estudo, profissionais de enfermagem de nível superior (enfermeiros) e nível médio (auxiliares e técnicos de enfermagem). Indicadores de estoque, composição, distribuição (por faixa etária e sexo) e razão de enfermeiros para médicos foram incluídos.

**Resultados:**

houve aumento no número de profissionais, entre 2005 e 2010, principalmente nos profissionais de nível médio e técnico. Há mais profissionais entre 36 e 55 anos, com predominância do sexo feminino em todas as categorias, apesar do aumento do sexo masculino. Observou-se distribuição desigual de profissionais nas regiões do país, sendo a região Sudeste a com maior número de profissionais. A razão de enfermeiros por médico é inferior a um nas regiões Sul e Sudeste.

**Conclusão:**

apesar do elevado contingente de enfermeiros, sua distribuição é desigual. O crescimento de técnicos de enfermagem superou significativamente o de enfermeiros, indicando políticas de formação técnica mais intensivas que as encontradas no ensino superior.

## Destaques

**(1)** A força de trabalho em enfermagem ainda apresenta uma distribuição desigual no Brasil.

**(2)** Há uma diferença na composição da equipe de enfermagem.

**(3)** Deve-se planejar a força de trabalho para garantir o cuidado à população.

## Introdução

A força de trabalho em saúde (FTS) é um componente fundamental nos sistemas e serviços de saúde. O desenvolvimento de trabalhadores de saúde capazes, motivados e apoiados é essencial para a prestação dos cuidados de saúde e para superar os obstáculos que impedem o alcance dos objetivos de saúde nacionais e globais. Contudo, estimativas da FTS global mostram escassez de 15 milhões de trabalhadores em 2020, com previsão de redução para 10 milhões até 2030^1^. Além da escassez, muitos países enfrentam grandes desafios em sua FTS como a inadequada combinação de habilidades e iniquidades na distribuição geográfica^2^.

A análise da situação atual da FTS por meio de dados confiáveis e pesquisas abrangentes é essencial para compreender melhor as forças que impulsionam a escassez, a combinação de habilidades e os desequilíbrios geográficos dos trabalhadores, assim como para o planejamento e desenvolvimento de políticas eficazes para lidar com essas questões^2^. Desse modo, o uso da evidência para o planejamento da FTS é de suma importância para a construção e consolidação de sistemas e serviços de saúde resilientes. Esta relevância vem sendo reconhecida e evidenciada face aos desafios enfrentados mundialmente em um cenário de emergência internacional como a pandemia de COVID-19^3^. Neste sentido, são imprescindíveis a vontade política e a cooperação internacional para o desenvolvimento e implementação de planos nacionais direcionados e alinhados ao uso de conhecimentos na construção e fortalecimento dos sistemas de saúde. Principalmente no contexto em que os formuladores de políticas e gestores são desafiados a assegurar a disponibilidade e acessibilidade à saúde para toda a população, considerando o aumento da demanda por profissionais de saúde em uma conjuntura de escassez e má distribuição destes profissionais^4^.

A literatura enfatiza a contribuição da força de trabalho de enfermagem e obstetrícia para a melhoria na saúde, utilizando um modelo de cuidado centrado nas pessoas, favorecendo a proximidade com os usuários dos serviços de saúde e comunidade. A enfermagem e a obstetrícia, em particular, representavam mais de 50% da escassez de trabalhadores da saúde em todo o mundo^5^ ^)-(^ ^6^.

A criação do Sistema Nacional de Saúde brasileiro, intitulado Sistema Único de Saúde (SUS), que estabeleceu o direito universal de acesso aos cuidados de saúde, causou a ampliação das políticas públicas, tanto em termos de acesso como cobertura da população. Apesar da expansão dos espaços de atuação dos profissionais de enfermagem, em especial no âmbito do SUS e na Atenção Primária à Saúde, atrair e reter esta força de trabalho representam enorme desafio para os gestores de saúde^7^ ^)-(^ ^8^. Neste contexto, a enfermagem brasileira, que compreendia aproximadamente 70% da FTS no país em 2019, totalizando mais de 2 milhões e 700 mil profissionais habilitados a exercer a profissão, e representando uma densidade de 12,97 profissionais por mil habitantes em 2022, tem um importante papel no sistema de saúde, muito além de sua representação numérica^4^ ^)-(^ ^9^.

Considerando que a dificuldade na implementação de políticas ou estratégias, bem como a falta de apoio político para o monitoramento de programas e políticas, são consideradas limitações significativas que refletem no processo de tomada de decisão no contexto brasileiro, este estudo apresentou como objetivo analisar a disponibilidade (estoque e composição) e acessibilidade (distribuição geográfica) da força de trabalho de enfermagem no Brasil. 

## Método

### Tipo de estudo

Trata-se de estudo descritivo e transversal com coleta retrospectiva de dados, que seguiu o guia STROBE.

### População do estudo

Foram coletadas informações relacionadas aos trabalhadores de enfermagem que compõem a equipe de enfermagem, ou seja, profissionais de enfermagem de nível superior (enfermeiros) e profissionais de nível médio (auxiliar de enfermagem e técnico de enfermagem). Conforme a Classificação Internacional Padrão de Ocupações (ISCO-08) de 2008: enfermeiro profissional (código ISCO 2221) e profissional associado de enfermagem (código ISCO 3221) equivalem à Classificação Brasileira de Ocupações (CBO): enfermeiros e equiparados (código CBO 2235) e técnicos e auxiliares de enfermagem (código CBO 3222), segundo a Lei do Exercício Profissional (Lei n.º 7.498 de 25 de junho de 1986), que dispõe sobre o exercício profissional de enfermagem no país. Em relação aos profissionais médicos, foi utilizado o ISCO-08 código 2211, e 2212 e código CBO 2002.

### Modelo teórico e indicadores de análise de recursos humanos em saúde

Para orientar o campo de estudo, o quadro conceitual fundamentado na sequência de quatro dimensões críticas: *Availability, Accessibility, Acceptability and Quality* (AAAQ)^10^ foi utilizado, o qual apresenta as dimensões disponibilidade, acessibilidade, qualidade e aceitabilidade aplicadas à FTS. O conceito de disponibilidade é apresentado como o suprimento suficiente e estoque de profissionais de saúde apropriados, com as competências relevantes, produção e combinação de habilidades que correspondam às necessidades de saúde da população. 

Entende-se como acessibilidade: a distribuição equitativa dos profissionais de saúde em termos de tempo de viagem e transporte (espacial); horário de funcionamento e presença da força de trabalho correspondente (temporal); atributos da infraestrutura (físicos, por exemplo, edifícios adaptados para deficientes); mecanismos de referência (organizacionais) e os recursos diretos e indiretos de custo dos serviços, tanto formais quanto informais (financeiros)^10^.

Para estruturar a coleta de dados, utilizou-se a ferramenta Contas Nacionais da Força de Trabalho em Saúde (CNFTS), desenvolvida pela Organização Mundial da Saúde (OMS) em sua versão em português (2020)^11^. A análise apresentada neste artigo enfocou a disponibilidade dos profissionais - quanto ao seu número (densidade em relação à população nacional) e composição (sexo, idade, nível de formação e proporção entre enfermeiros e médicos); e acessibilidade-espacial (distribuição por região), nas desagregações por categoria, segundo a Figura 1.

**Figura 1:** Indicadores das Contas Nacionais da Força de Trabalho em Saúde e complementares selecionados para a coleta e análise das informações

Elemento do quatro conceitual AAAQ*	Área do indicador	Nome do indicador	Descrição utilizada
Disponibilidade	Estoque ativo da força de trabalho em saúde	Densidade dos profissionais de saúde	Numerador: Número de profissionais de saúde, definido em número de pessoas. Denominador: População total.
Disponibilidade	Distribuição	Distribuição dos profissionais por faixa etária	Numerador: Número de profissionais de saúde ativos em uma faixa etária específica. Denominador: Número total de profissionais de saúde ativos, definido em número de pessoas.
Acessibilidade	Estoque ativo da força de trabalho em saúde	Densidade dos profissionais no nível subnacional - regional	Numerador: Número de profissionais de saúde ativos em unidades administrativas subnacionais, definidos em número de pessoas. Denominador: População total em nível subnacional.
Disponibilidade	Complementar	Razão de profissionais de enfermagem por médicos - nacional e regional e por categoria.	Numerador: Número total de enfermeiros/profissionais de enfermagem habilitados à prática. Denominador: Número total de médicos habilitados à prática^†^.

*A estrutura AAAQ é uma sequência de quatro dimensões críticas para analisar: Disponibilidade; Acessibilidade; Aceitabilidade e Qualidade (do inglês *Availability, Accessibility, Acceptability and Quality*); †Conforme a Organização para a Cooperação e Desenvolvimento Econômico (OECD)^12^, considera-se como profissionais habilitados à prática aqueles que têm requisitos de formação e qualificação e licença (registro/habilitação) para o exercício da profissão. O conceito de profissionais ativos se refere aos que têm formação e habilitação como pré-requisitos para prestação de serviços a pacientes e comunidades, gestão ou ensino. Já os profissionais em exercício são aqueles que prestam serviços diretos a pacientes e comunidades

### Coleta e análise de dados

Os dados foram identificados por meio de uma combinação de base de dados disponibilizadas em sites institucionais: a) Plataforma Rede Interagencial de Informações para a Saúde (RIPSA)^13^, rede composta por instituições governamentais e não governamentais^14^: quantitativo de profissionais aptos à atuação (auxiliares, técnicos e enfermeiros) por sexo e por estado nos anos 1990, 1995 e 2007 e quantitativo de profissionais (auxiliares, técnicos e enfermeiros) inscritos por estado nos anos de 1990 a 2008 e 2010; b) Conselho Federal de Enfermagem (Cofen)^9^, órgão responsável pela regulação das profissões relacionadas à enfermagem no país, que agrega dados dos Conselho Regional de Enfermagem (Coren) por meio do sistema Cofen/Coren: número de profissionais aptos à atuação por estado, categoria, idade e sexo dos profissionais registrados; c) Contas Nacionais da Força de Trabalho em Saúde (CNFTS)^15^, desenvolvida pela Organização Mundial da Saúde (OMS), que tem como base o quadro conceitual do mercado de trabalho em saúde^11^: dados sobre sexo, idade dos profissionais de enfermagem para os anos de 2014, 2017, 2019, podendo variar entre habilitados a prática e em exercício; e d) Instituto Brasileiro de Geografia e Estatística (IBGE)^16^, principal provedor de dados e informações do país, atendendo necessidades de diversos segmentos da sociedade civil e de órgãos governamentais^17^: os dados referentes à população brasileira, por unidade federativa e por região, foram obtidos por meio das estimativas populacionais do IBGE publicados no Diário Oficial da União.

Embora a maioria dos indicadores propostos considere apenas os profissionais ativos, o nível de atividade dos profissionais utilizados nas análises variou conforme informação disponível nas bases de dados. Também foi realizada coleta de informação disponível em relatórios publicados como: a) Relatório Integrado de Gestão do Conselho Federal de Enfermagem de 2019^18^ *:* apresenta o quantitativo e sexo dos profissionais - enfermeiros e auxiliares e técnicos de enfermagem - por estado para o ano de 2019; b) Relatório Comissão de Business Intelligence Produto 2^19^: exibe dados dos profissionais de enfermagem inscritos nos Conselhos Regionais de Enfermagem (Coren) nos estados por categoria profissional, sexo e faixa etária no ano de 2010*;* c) Relatório Comissão de *Business Intelligence* ^20^: com dados dos profissionais de enfermagem inscritos nos CORENs nos estados por categoria profissional, sexo e faixa etária no ano de 2011, e d) Perfil da Enfermagem no Brasil (Relatório Final)^21^ *,* resultante do projeto de pesquisa cujo objetivo central foi traçar o perfil da equipe de enfermagem no Brasil. No respectivo documento, foram identificados os profissionais de enfermagem segundo faixa etária em 2013.

A coleta de dados ocorreu no período de dezembro de 2022 a abril de 2023 por dois pesquisadores simultaneamente. Apesar de os autores seguirem o mesmo método de busca de dados para cada base supracitada, identificou-se ausência de informações, o que impossibilitou a coleta sequencial de todos os anos, em todas as bases de dados utilizadas. 

Os dados encontrados foram organizados no aplicativo Excel e realizados cálculos dos indicadores descritos na Figura 1, considerando, além do aumento de percentual (taxa de crescimento), a média de profissionais por sexo, por categoria (enfermeiros, técnicos e auxiliares de enfermagem) e por idade.

No que diz respeito ao Comitê de Ética, apesar de ser um estudo descritivo, este artigo integra estudo mais abrangente, que foi submetido e aprovado pelo Comitê de Ética da Escola de Enfermagem de Ribeirão Preto (CAAE: 43184621.0.0000.5393 e Número do Parecer: 4.859.633).

## Resultados

### Disponibilidade da força de trabalho

O número total de registros profissionais de enfermagem no sistema Conselho Federal de Enfermagem (Cofen) e Conselho Regional de Enfermagem (Coren) era de 2.767.741 em dezembro de 2022^9^. Ao analisar a sua composição, foi possível identificar que é majoritariamente formada por profissionais de enfermagem de nível médio e técnicos, 75,47% (totalizando 2.088.700 técnicos e auxiliares de enfermagem) e minoritariamente 24,53% de nível superior (679.041 enfermeiros). Em relação à densidade de profissionais de enfermagem por mil habitantes no país, verificamos expressivo aumento entre 1990 e 2022, crescendo de 0,96 para 12,97, respectivamente. Apesar de este aumento ser constante e observado ao longo dos anos, é bem expressivo entre o período de 2005 a 2010, quando a densidade de profissionais no país praticamente dobrou em cinco anos, indo de 3,82 para 7,58, respectivamente. Parte responsável por esse aumento foram os profissionais de nível médio, passando de 0,94 para 3,24 os técnicos de enfermagem, e de 2,33 para 2,80 os auxiliares neste período. Outro momento a ser observado é o período de 2019 a 2022, o qual perpassa a pandemia, em que o crescimento da densidade dos profissionais em três anos é de quase 22% (de 10,64 para 12,97), enquanto no período anterior, o crescimento foi de aproximadamente 14% (de 9,35 para 10,64), como ilustra a Figura 2.

**Figura 2:** Evolução do número e da densidade de profissionais de enfermagem por mil habitantes. Brasil, 1990-2022

Fonte: Número de registros de profissionais de enfermagem por categoria: Conselho Federal de Enfermagem (Cofen)^9^ e Rede Interagencial de Informações para a Saúde (RIPSA)^13^. Dados da população obtidos pelo Instituto Brasileiro de Geografia e Estatística (IBGE)^16^

Quanto à caracterização dos profissionais, ao analisar a distribuição dos profissionais de enfermagem (sem distinção de categorias) por sexo, nota-se que, apesar de um leve aumento no número de profissionais masculinos, as mulheres continuaram a predominar nas últimas duas décadas, de 1990 a 2019, representando 88,00% em 2019, conforme indicado na Figura 3, subitem (a). Análise mais detalhada das categorias profissionais no período de 1990 a 2011 revela aumento ligeiramente mais pronunciado no contingente masculino entre os profissionais de nível superior, passando de 6,42% em 1990 para 11,86% em 2011, conforme ilustrado na Figura 3, subitem (b).

**Figura 3:** Caracterização da força de trabalho de enfermagem por sexo, total e por categoria. Brasil, 1990-2019

Fonte: Número de registros profissionais de enfermagem, 2011: Conselho Federal de Enfermagem, Cofen^20^; 1990 a 2010: Rede Interagencial de Informações para a Saúde (RIPSA)^13^ e; 2014 a 2019: Contas Nacionais da Força de Trabalho em Saúde (CNFTS)^15^; Número de registros de profissionais de enfermagem por categoria e por sexo: Conselho Federal de Enfermagem (Cofen)^19^ ^)-(^ ^20^ e Rede Interagencial de Informações para a Saúde (RIPSA)^13^

Durante o período de 2010 a 2019, ao analisarmos a distribuição etária dos profissionais de enfermagem, identificou-se que, apesar de os dados serem provenientes de diferentes fontes, houve predominância de indivíduos entre 36 e 55 anos. Entretanto, nos anos de 2017 e 2019, observa-se diminuição no percentual de profissionais com menos de 35 anos. Os dados mostram que os profissionais com menos de 35 anos representavam mais de 35% em todos os anos estudados, variando de 35,70% a 45,90%, enquanto aqueles com 56 anos ou mais representavam mais de 6% do total de profissionais de enfermagem, com percentuais entre 6,09% e 9,81%. Estas informações foram obtidas a partir do número de registros de profissionais de enfermagem por categoria para os anos de 2010 e 2011^9^, para 2013^21^ e, para 2014, 2017 e 2019, extraídos das CNFTS^15^.

Para analisar a combinação de habilidades, utilizaram-se a densidade e a razão entre os profissionais de enfermagem e medicina no país, apresentam-se as Tabelas 1 e 2. Quando comparamos os dados da densidade de enfermeiros aos de médicos por 10 mil habitantes para o ano de 2022, esta se apresentava ligeiramente maior para os enfermeiros (31,83 e 27,38 respectivamente), atingindo uma razão nacional de 1,16 enfermeiros por médico no mesmo ano. A maior diferença entre as densidades destes profissionais pode ser vista nas regiões Norte e Nordeste, que apresentaram também as maiores razões de enfermeiros por médicos no país (1,98 e 1,64 respectivamente). No outro extremo, encontram-se as regiões Sul e Sudeste que apresentaram as menores razões de enfermeiro por médico (0,95 e 0,99 respectivamente). Ao analisar a razão de profissionais de enfermagem por médicos, foi identificada uma razão nacional de 4,74, variando entre 3,83 profissionais de enfermagem por médico na região Sul e 8,52 na região Norte.

**Tabela 1:** Densidade de profissionais, total e por categoria, por 10.000 habitantes e razão de profissionais de enfermagem, total e enfermeiros, por médico. Brasil, 2022

Região	Densidade por 10 mil habitantes							Razão	
	ENF^†^	Téc Enf^‡^	Aux Enf^§^	Prof Enf^ǁ^	Médicos*	ENF^†^ e médicos	Prof Enf^ǁ^ e médicos	ENF^†^ por médico	Prof Enf^ǁ^ por médico
Norte	28.72	85.65	9.29	123.66	14.52	43.21	138.18	1.98	8.52
Nordeste	31.64	72.40	11.71	115.75	19.29	50.92	135.04	1.64	6.00
Sudeste	33.56	78.67	34.87	147.10	33.90	67.46	181.00	0.99	4.34
Sul	26.80	73.22	13.07	113.09	29.52	56.32	142.61	0.95	3.83
Centro-Oeste	35.91	78.39	7.89	122.19	31.02	66.93	143.21	1.16	3.94
Total	31.83	76.79	21.12	129.75	27.38	59.22	157.13	1.16	4.74

Fonte: Número de registros de profissionais de enfermagem por categoria: Conselho Federal de Enfermagem (Cofen)^9^; número de habitantes (população brasileira) em 2022: Instituto Brasileiro de Geografia e Estatística (IBGE)^16^ e número de registros de profissionais médicos: Demografia Médica no Brasil 2023^22^

*Foi utilizado o número de registros de profissionais de medicina para possibilitar a comparação com os dados da enfermagem; ^†^ENF = Enfermeiros; ^‡^Téc Enf = Técnicos de enfermagem; ^§^Aux Enf = Auxiliares de enfermagem; ^ǁ^Prof Enf = Profissionais de enfermagem

**Tabela 2:** Número de profissionais de enfermagem, total e por categoria, e taxa de crescimento por região. Brasil, 2000 e 2022

Região	ENF*			Téc Enf^†^			Aux Enf^‡^			Prof Enf^§^		
	2000	2022	Taxa	2000	2022	Taxa	2000	2022	Taxa	2000	2022	Taxa
Norte	4.150	54.305	1209%	3.408	161.936	4652%	13.683	17.559	28%	21.241	233.800	1001%
Nordeste	17.959	182.442	916%	10.912	417.526	3726%	52.550	67.544	29%	81.421	667.512	720%
Sudeste	38.054	300.804	690%	44.627	705.131	1480%	185.621	312.521	68%	268.302	1.318.456	391%
Sul	9.263	81.487	780%	8.822	222.594	2423%	42.059	39.746	-5%	60.144	343.827	472%
Centro-Oeste	4.695	60.003	1178%	10.074	130.965	1200%	16.254	13.178	-19%	31.023	204.146	558%
Total	74.121	679.041	816%	77.843	1.638.152	2004%	310.167	450.548	45%	462.131	2.767.741	499%

Fonte: Números de profissionais de enfermagem registrados por categoria para o ano de 2022, obtidos por meio do Conselho Federal de Enfermagem (Cofen)^9^ e para o ano de 2000, obtidos por meio da Rede Interagencial de Informações para a Saúde (RIPSA)^13^

*ENF = Enfermeiros; ^†^Téc Enf = Técnicos de enfermagem; ^‡^Aux Enf = Auxiliares de enfermagem; ^§^Prof Enf = Profissionais de enfermagem

Ao analisar a disponibilidade do grupo de profissionais no país, conforme Tabela 1, notou-se densidade nacional de 59,22 enfermeiros e médicos, bem como 57,13 profissionais de enfermagem e médicos por 10 mil habitantes. Além disso, foi possível identificar a menor densidade no país, apresentada pelas regiões Norte e Nordeste, tanto em relação aos profissionais com formação superior (43,21 e 50,92 enfermeiros e médicos por 10 mil habitantes, respectivamente), e quanto aos profissionais de nível médio/técnico (138,18 e 135,04 profissionais de enfermagem e médicos por 10 mil habitantes).

### Acessibilidade da força de trabalho

Ao analisar a distribuição dos profissionais de enfermagem entre as regiões do país, pode-se observar que o maior quantitativo está concentrado na região Sudeste, seguida pela região Nordeste, notando-se a mesma distribuição nas diferentes categorias profissionais. A região Norte apresenta-se como a região que tem a menor quantidade de enfermeiros no país, porém, quando analisados o número de profissionais de enfermagem, auxiliar e técnico de enfermagem, a região que possui a menor representatividade é a Centro-Oeste, como destacado na Tabela 2.

Apesar de a região Sudeste ter a maior representatividade do país em relação ao quantitativo de profissionais de enfermagem em suas diferentes categorias, foi a que apresentou o menor crescimento durante o período de 2000 a 2022, considerando o total de profissionais de enfermagem e enfermeiros. A região Norte foi a região com maior crescimento em relação às diferentes categorias analisadas, exceto para auxiliares de enfermagem. Já, quando observados apenas os auxiliares de enfermagem, as regiões Sul e Centro-Oeste apresentaram um decréscimo na categoria, como mostra a Tabela 2. 

Dessa forma, é relevante destacar a densidade de profissionais, ou seja, a quantidade de profissionais por habitantes em um determinado local. Em relação a este indicador, a região Sudeste, apesar de apresentar a menor taxa de crescimento, no período analisado por este estudo, é a região com maior densidade de profissionais de enfermagem do país (e quantitativo de profissionais), uma vez que possui a segunda maior densidade de enfermeiros e a maior densidade de profissionais de nível médio e técnico, com especial participação do auxiliar de enfermagem (34,87 em contraste ao cenário de desaceleração desta categoria). Quando observada a densidade de enfermeiros, a região Centro-Oeste é a primeira, mesmo apresentando o quarto menor número de enfermeiros dentre todas as regiões. 

A região Nordeste, apesar de se destacar pelo quantitativo de profissionais de enfermagem, estando na segunda posição em todas as categorias, possui a segunda menor densidade de profissionais de enfermagem e a menor densidade de técnicos e auxiliares em conjunto. O grande crescimento da região Norte ainda não se reflete em melhores densidades de profissionais, representando a segunda menor densidade nacional. A região Sul apresenta a menor densidade de profissionais de enfermagem e enfermeiros no país e a segunda maior densidade de auxiliares de enfermagem, conforme Tabela 1.

## Discussão

A densidade da enfermagem no país, de aproximadamente 130 profissionais de enfermagem e 32 enfermeiros por 10 mil habitantes em 2022, foi identificada como superior à média dos países da Região da América Latina e do Caribe (ALC) e da Organização para a Cooperação e Desenvolvimento Econômico (OCDE), que apresentam um pouco mais de 35 e 103 profissionais de enfermagem por 10 mil habitantes respectivamente para o mesmo ano (informação disponível para ALC de 33 países, e OCDE para 38 países)^23^, mostrando um aparente “excesso de profissionais” no Brasil. É importante destacar que os dados globais podem estar considerando os profissionais em exercício e/ou os ativos, enquanto os dados analisados do Brasil referem-se aos profissionais habilitados à prática, considerando o número de inscrições no Sistema Cofen/Coren. 

Adicionalmente, no Brasil, em 2022, sete em cada dez profissionais de enfermagem possuem nível médio e/ou técnicos (75%), sendo um cenário oposto ao mundial, em que 69% dos profissionais possuem nível superior (considerando dados em anos anteriores). Na região da Europa, enfermeiros compõem 70% da força de trabalho de enfermagem (por exemplo, Reino Unido, com 80% dos profissionais com formação superior)^24^. Com exceção de alguns países, como Canadá e Estados Unidos (75.46% e 85,74% dos profissionais com formação superior respectivamente)^15^ ^),(^ ^24^, a região das Américas tem sua força de trabalho de enfermagem composta majoritariamente por profissionais de nível médio e/ou técnicos, contudo, em uma proporção inferior ao Brasil (59%)^25^.

Durante o período analisado (1990 a 2022), foram identificados dois períodos de aumento significativo no número de profissionais de enfermagem no Brasil. O primeiro ocorreu entre 2005 e 2010, quando houve um aumento expressivo na densidade de profissionais, quase dobrando de 3,82 para 7,58. Os técnicos de enfermagem desempenharam papel fundamental nesse aumento, passando de 0,94 para 3,24. Essa expansão foi impulsionada por estratégias nacionais, como o Projeto de Profissionalização dos Trabalhadores da Área da Enfermagem (PROFAE)^26^. O segundo período de crescimento na densidade de profissionais de enfermagem, aproximadamente 22% entre 2019 e 2022, contradiz parcialmente os resultados de uma revisão integrativa internacional que examinou 43 estudos (pré e pós-COVID-19) e indicou aumento significativo na taxa de intenção de abandono da profissão após a pandemia de COVID-19, conforme também observado pelo International Council of Nurses (ICN). Dessa forma, pesquisa com as Associações Nacionais de Enfermagem apontou aumento no número de enfermeiros deixando a profissão. Nesse contexto, enfatizam-se como fatores relacionados a esta intenção e/ou abandono: carga de trabalho pesada, recursos insuficientes, esgotamento físico e estresse^27^ ^)-(^ ^28^. Assim sendo, ressalta-se a imperatividade de examinar tal cenário no país, analisando não apenas a quantidade de profissionais envolvidos no cuidado direto aos pacientes e à comunidade, mas também o número de indivíduos que optaram por abandonar essa nobre profissão.

Apesar de ser uma profissão jovem, com mais de um terço dos profissionais em início de carreira e menos de 9% próximos à aposentadoria (2019), esboça-se um envelhecimento da profissão no país (2010 a 2019). A força de trabalho de enfermagem mais jovem pode ajudar a garantir a sustentabilidade da profissão de enfermagem. À medida que os enfermeiros com mais idade se aposentam, é importante ter uma nova geração para substituí-los e continuar a prestar cuidados de alta qualidade^29^. Apesar de estar distante do cenário de alguns países das Américas, com populações substancialmente mais velhas, como o Panamá e República Dominicana (com mais de 26% dos profissionais com 55 anos ou mais)^15^, e países da Europa, por exemplo, Bulgária, Islândia e República da Moldávia (com aproximadamente 40% dos profissionais com 55 anos ou mais)^30^, esta informação sugere a necessidade de monitorar este indicador e realizar análises entre as diferentes categorias, a fim de melhor compreender os fluxos de entrada e saída e as necessidades dos profissionais.

Em relação à característica da força de trabalho de enfermagem, nota-se perfil predominantemente de profissionais do sexo feminino. Neste estudo foi possível identificar um aumento no contingente masculino, representando 12% dos profissionais habilitados à prática em 2019, incremento ligeiramente maior na categoria de enfermeiros (de 6% para 12% 1990 -2011). Dados referentes aos profissionais de enfermagem em exercício apontam percentual de 13,7% no ano de 2021 para o país^16^. Estes valores são próximos aos identificados em outros países da região das Américas, com 13% em 2019^16^, destacando-se a Argentina com aproximadamente 20% dos profissionais de enfermagem do sexo masculino em 2020^15^ ^),(^ ^25^.

Embora os profissionais de saúde do sexo feminino sejam responsáveis pela prestação da maioria dos cuidados em todos os níveis e ambientes, ao representarem aproximadamente 67% dos trabalhadores assalariados no setor de saúde, encontram barreiras no trabalho. Tais barreiras não são enfrentadas pelos profissionais do sexo masculino, como o preconceito, discriminação, assédio sexual e violência, adicionalmente aos riscos desproporcionais que surgiram durante a pandemia da COVID-19^31^. A integração do sexo e idade nas estratégias da FTS é necessária para garantir que sejam adotadas abordagens sensíveis e baseadas em evidências para o planejamento e gestão dos profissionais. A falta de dados precisos sobre a composição por sexo e idade em diversos países impede o aprimoramento das condições de trabalho e das estruturas de carreira para apoiar a participação e progresso das mulheres na FTS, bem como a valorização das contribuições das mulheres e seus profissionais nos sistemas de cuidados de saúde^32^. 

Ao estimar a combinação de habilidades da FTS por meio da comparação da razão de profissionais, são obtidas informações sobre a possibilidade de ganhos de eficiência e também a razão de enfermeiros para médicos como potencial indicador de qualidade do atendimento ao usuário^2^ ^),(^ ^33^. O Brasil apresentou razão nacional em 2022 de 1,16 enfermeiros e duas de suas regiões, regiões Sul e Sudeste, apresentaram razões de enfermeiro por médico inferiores a 1 (0,95 e 0,99 respectivamente). Estas foram identificadas como inferiores à média dos países da ALC e da OCDE, que correspondem a 1,9 e 2,7 enfermeiros para cada médico, respectivamente para o mesmo ano (informação disponível para ALC de 32)^23^. Vários países da região apresentam índices acima desse valor, incluindo a Dominica, o mais alto da região, com 5,5 enfermeiros por médico. Por outro lado, destaca-se a proporção de 0,6 enfermeiros por médico em Colômbia, no extremo mais baixo da região ALC.

Este estudo identificou uma razão de profissionais de enfermagem por médicos nacional de 4,74 em 2022. Ao considerar a média da OCDE e da ALC, esta razão se apresenta relativamente maior no Brasil. Outrossim, é importante reiterar que a força de trabalho de enfermagem tem características diferentes (composta majoritariamente por profissionais de nível médio e técnico). Ressalta-se, ainda, que o documento da OCDE apresenta dados dos profissionais ativos no Brasil em 2019 (3,4 profissionais de enfermagem) e identifica valor superior ao destes grupos de países (a média da OCDE é de 2,6 e ALC, de 1,6)^34^. Dessa maneira, é fundamental repensar a combinação de habilidades profissionais de saúde no contexto de envelhecimento da população e aumento da carga de doenças crônicas no país.

Ao considerar a acessibilidade à força de trabalho, as estimativas de densidade informam a disponibilidade per capita de profissionais de saúde, assim como a variação da densidade entre as regiões geográficas fornece informações sobre a distribuição equitativa dos profissionais de saúde entre regiões^2^. O documento de base para a Estratégia Global de Recursos Humanos em Saúde: Força de trabalho 2030 da OMS estimou o “limite mínimo” de 44,50 médicos, enfermeiros e parteiras por 10 mil habitantes como necessário para oferecer uma cobertura universal de saúde à população^35^ ^)-(^ ^36^. Ao analisar a disponibilidade do grupo de profissionais no país, este estudo observou densidade nacional de 59,22 enfermeiros e médicos por 10 mil habitantes, incluindo profissionais de nível médio e técnicos de 157,13 profissionais de enfermagem e médicos, ou seja, ambas as densidades nacionais alcançam este “limite mínimo”. Ao considerar somente os profissionais com formação superior, a região Norte apresentou densidade inferior a este limite estabelecido (43,21 enfermeiros e médicos por 10 mil habitantes). Entretanto, embora esse limite seja uma métrica valiosa para comparações internacionais e monitoramento, não é aconselhável utilizá-lo diretamente para estabelecer metas nacionais. Tal abordagem desconsidera as peculiaridades do país, tais como condições de saúde, estruturas e a presença de outros grupos ocupacionais diferentes das três profissões existentes no país. Portanto, é fundamental desenvolver metas adaptadas ao contexto nacional, considerando as prioridades políticas para a disponibilidade e composição da Força de Trabalho em Saúde (FTS)^2^.

Nas duas últimas décadas, houve um movimento de expansão da formação de Enfermagem no Brasil, com a criação de escolas em todas as regiões. Esse movimento resultou da democratização do acesso ao ensino superior e tem como consequência mais disponibilidade de enfermeiros no mercado^37^. Apesar desta expansão, as Escolas de Enfermagem se concentram nos estados de maior densidade populacional e de concentração de renda do país, acompanhando a distribuição do Produto Interno Bruto (PIB) e as desigualdades econômicas e sociais das regiões brasileiras^37^.

Os dados identificados neste estudo, para o ano de 2022, indicam que somente a região Sudeste apresentou quase 50% do número de profissionais de enfermagem do país. Nesse sentido, esta região também apresentou maiores números de profissionais em anos anteriores, com base em estudo que analisou a informação para enfermeiros em diferentes anos da década de 2010^38^. As áreas remotas podem enfrentar a privação de profissionais qualificados, prolongando as disparidades regionais. Em contraste, as áreas centrais e ricas podem ter menor oferta de profissionais qualificados e menor empregabilidade^39^.

Estudo buscou estimar o número de enfermeiros empregados que deveriam ser redistribuídos para que houvesse uma distribuição geográfica mais adequada desses profissionais no Brasil, e identificou que a região Sudeste deveria redistribuir 8,8% dos seus enfermeiros entre as regiões brasileiras, representando aproximadamente 6 mil empregos de enfermeiros a serem redistribuídos para o alcance de uma situação de igualdade em relação a essa razão^40^. Ainda, análise sobre a cobertura populacional de enfermeiros, nos estados da região Norte e Nordeste, mostrou cobertura populacional abaixo de 5 enfermeiros para cada 10 mil habitantes, com valores acima de 10 enfermeiros a cada 10 mil habitantes^38^. 

As limitações deste estudo são referentes às características de cada base de dados consultada, dada a fragmentação da informação, e do uso de diferentes metodologias na coleta de dados, assim como nível de atuação profissional. Destaca-se a necessidade de padronização na coleta e divulgação de dados de profissionais de saúde. 

O monitoramento e a avaliação contínuos da força de trabalho de enfermagem são fundamentais. Nesse sentido, considerando as distorções concernentes à distribuição desigual do contingente de profissionais enfermeiros nos estados brasileiros, é relevante que o poder público seja o responsável por esse processo, ofertando cursos de enfermagem adequados às realidades locais e às exigências de formação globais, nos territórios vazios de enfermeiros, mormente no interior do Brasil^41^.

Em suma, este estudo possui papel significativo no avanço do conhecimento científico, fornecendo análise detalhada da disponibilidade e acessibilidade da força de trabalho de enfermagem no Brasil. Ao empregar, juntamente, métodos descritivos e transversais e indicadores criteriosamente selecionados, os pesquisadores conseguiram traçar um panorama abrangente da situação. Os resultados destacam mudanças ao longo do tempo, revelando aumento notável no número de profissionais de enfermagem, especialmente no nível médio e técnico, entre 2005 e 2010. Além disso, a distribuição desigual dos profissionais em diferentes regiões do país foi identificada, com a região Sudeste se destacando em termos de quantidade. A análise também apontou desafios persistentes, como a predominância do sexo feminino em todas as categorias e a necessidade de políticas públicas mais robustas para diversificar e redistribuir os profissionais em todo o Brasil. Essas descobertas não apenas informam decisões futuras nas áreas de saúde e educação, mas também oferecem insights valiosos para a formulação de estratégias que visem melhorar a qualidade dos cuidados de saúde e promover a equidade no acesso aos serviços médicos em todo o país.

Nesse sentido, enfatiza-se a importância de políticas públicas que considerem as estatísticas para o planejamento e execução de políticas e estratégias, de modo a reconfigurar a distribuição de profissionais para promover uma alocação mais equitativa em todas as regiões do território brasileiro. Destaca-se o esforço realizado com o Programa de Valorização dos Profissionais da Atenção Básica (PROVAB), que objetivou prover equipes da Atenção Básica e da Estratégia Saúde da Família com enfermeiros, dentistas e médicos em áreas remotas e de maior vulnerabilidade. 

Por último, destaca-se a necessidade premente de novas pesquisas e políticas que examinem profundamente a eficácia da integração dos profissionais em suas funções e locais de atuação. Isso é crucial para compreender a crescente demanda por profissionais de enfermagem, uma necessidade que parece se intensificar a cada ano, apesar do aumento numérico desses profissionais.

## Conclusão

Apesar do grande número de profissionais de enfermagem, há disparidade em sua distribuição em todo território nacional. Ainda, é possível notar que os enfermeiros, além de estarem em menores quantidades, apresentaram crescimento bem menos acentuado do que os técnicos de enfermagem em algumas regiões do país, assim, o crescimento de técnicos de enfermagem superou o de enfermeiros, indicando políticas de formação técnica mais intensivas que no ensino superior.

# Translation: Número, composición y distribución de la fuerza de trabajo de enfermería en Brasil: una fotografía*

**DOI:** 10.1590/1518-8345.6937.4286

**Authors:** Ana Paula Cavalcante de Oliveira, Ana Beatriz Zanardo Mion, Mariana Lopes Galante, Gabriela Di Donato, Carla Aparecida Arena Ventura

**Affiliations:** 1 Universidade de São Paulo, Escola de Enfermagem de Ribeirão Preto, Centro Colaborador de la OPS/OMS para el Desarrollo de la Investigación en Enfermería, Ribeirão Preto, SP, Brasil.

**Keywords:** Recursos Humanos en Salud, Enfermería, Organización y Administración, Políticas, Planificación y Administración en Salud, Brasil, Política Pública

## Abstract

**Objetivo:**

analizar la disponibilidad (en términos de número y composición) y accesibilidad (en términos de distribución geográfica) de la fuerza de trabajo de enfermería en Brasil.

**Método:**

estudio descriptivo y transversal, con recolección retrospectiva de datos, identificados a través de una combinación de bases de datos disponibles en sitios *web* institucionales y estructurados en función de los indicadores de las “Cuentas Nacionales del Personal de Salud” de la Organización Mundial de la Salud. La población del estudio fueron profesionales de enfermería de nivel superior (enfermeros) y nivel medio (auxiliares y técnicos en enfermería). Se incluyeron indicadores de número, composición, distribución (por franja etaria y sexo) y razón entre enfermeros y médicos.

**Resultados:**

aumentó el número de personal entre 2005 y 2010, principalmente la de nivel medio y técnico. La mayoría del personal tiene entre 36 y 55 años y predomina el sexo femenino en todas las categorías, pese a que aumentó la cantidad de trabajadores do sexo masculino. Se observó que la distribución de personal en las diferentes regiones de Brasil era desigual y la región Sudeste es la que tiene la mayor cantidad de profesionales. La proporción de enfermeros por médico es inferior a uno en las regiones Sur y Sudeste.

**Conclusión:**

a pesar de que la cantidad de enfermeros es elevada, la distribución es desigual. El aumento de la cantidad de técnicos en enfermería superó significativamente al de enfermeros, lo que indica que las políticas de formación técnica son más intensivas que las observadas en la educación superior.

## Destacados

**(1)** La distribución de la fuerza de trabajo de enfermería en Brasil es desigual.

**(2)** Hay una diferencia en la composición del equipo de enfermería.

**(3)** Hay que planificar la fuerza de trabajo para garantizar el cuidado de la población.

## Introducción

La fuerza de trabajo en salud (FTS) es un componente fundamental de los sistemas y servicios de salud. La formación de trabajadores sanitarios capaces, motivados y apoyados es esencial para brindar atención en salud y para superar los obstáculos que impiden lograr los objetivos de salud nacionales y mundiales. Sin embargo, las estimativas sobre la FTS mundial muestran que hay una escasez de 15 millones de trabajadores en 2020 y que se prevé que se reducirá a 10 millones para 2030^1^. Además de la escasez, muchos países enfrentan desafíos importantes con respecto a su FTS, como la combinación inadecuada de habilidades y desigualdades en la distribución geográfica^2^.

Analizar el estado actual de la FTS a través de datos confiables e investigaciones exhaustivas es fundamental para comprender mejor las fuerzas que impulsan la escasez, la combinación de habilidades y los desequilibrios geográficos de trabajadores, así como también para planificar y desarrollar políticas efectivas que aborden esos problemas^2^. Por lo tanto, es muy importante utilizar la evidencia para planificar la FTS a fin de construir y consolidar sistemas y servicios de salud resilientes. Esa relevancia se reconoció y se manifestó debido a los desafíos que se enfrentaron a nivel mundial en un escenario de emergencia internacional como fue el de la pandemia de COVID-19^3^. Por ende, la voluntad política y la cooperación internacional son esenciales para desarrollar e implementar planes nacionales orientados y basados en el uso del conocimiento para la construcción y el fortalecimiento de los sistemas de salud. Principalmente cuando los responsables de crear políticas y los gestores se ven desafiados a garantizar la disponibilidad y el acceso a la atención en salud para toda la población, considerando el aumento de la demanda de profesionales sanitarios en una situación de escasez y mala distribución de dichos profesionales^4^.

La literatura destaca la contribución de la fuerza de trabajo de enfermería y obstetricia para mejorar la salud, que utiliza un modelo de atención centrado en las personas, que favorece la cercanía con los usuarios de los servicios de salud y con la comunidad. La enfermería y la obstetricia, en particular, representaban más del 50% de la escasez de trabajadores de la salud en todo el mundo^5^ ^)-(^ ^6^.

La creación del Sistema Nacional de Salud brasileño, llamado Sistema Único de Salud (SUS), que estableció el derecho universal de acceso a la atención de salud, provocó la ampliación de las políticas públicas, tanto en términos de acceso como de cobertura de la población. Si bien ampliaron los espacios de trabajo de los profesionales de enfermería, especialmente en el ámbito del SUS y de la Atención Primaria de Salud, atraer y retener esa fuerza de trabajo representa un enorme desafío para los gestores de salud^7^ ^)-(^ ^8^. En este contexto, la enfermería brasileña, que representaba aproximadamente el 70% de la FTS del país en 2019, más de 2 millones 700 mil profesionales calificados para ejercer la profesión, es decir, una densidad de 12,97 profesionales por cada mil habitantes en 2022, tiene un papel importante en el sistema de salud, mucho más allá de su representación numérica^4^ ^)-(^ ^9^.

Dado que, tanto la dificultad para implementar políticas o estrategias, como la falta de apoyo político para los programas y políticas de seguimiento, se consideran limitaciones significativas que se reflejan el proceso de toma de decisiones en el contexto brasileño, este estudio tuvo como objetivo analizar la disponibilidad (en términos de número y composición) y accesibilidad (en términos de distribución geográfica) de la fuerza trabajo de enfermería en Brasil. 

## Método

### Tipo de estudio

Se trata de un estudio descriptivo, transversal, con recolección retrospectiva de datos, que siguió la guía STROBE.

### Población del estudio

Se recopiló información relacionada con los trabajadores de enfermería que integran el equipo de enfermería, es decir, profesionales de enfermería de nivel superior (enfermeros) y profesionales de nivel medio (auxiliares de enfermería y técnicos en enfermería). Según la Clasificación Internacional Uniforme de Ocupaciones (CIUO-08) de 2008: enfermero profesional (código CIUO 2221) y profesional asociado de enfermería (código CIUO 3221) equivalen a la Clasificación Brasileña de Ocupaciones (CBO): enfermeros y asimilados (código CBO 2235) y técnicos y auxiliares de enfermería (código CBO 3222), según la Ley de Ejercicio Profesional (Ley n.º 7.498, del 25 de junio de 1986), que regula el ejercicio profesional de la enfermería en Brasil. A los profesionales médicos les corresponde el CIUO-08 código 2211, y 2212 y el código CBO 2002.

### Modelo teórico e indicadores para el análisis de recursos humanos en salud

Para orientar el campo de estudio se utilizó el marco conceptual basado en la secuencia de cuatro dimensiones críticas: *Availability, Accessibility, Acceptability and Quality* (AAAQ)^10^, que presenta las dimensiones disponibilidad, accesibilidad, calidad y aceptabilidad aplicadas a la FTS. El concepto de disponibilidad se presenta como el suministro adecuado y la presencia de profesionales de salud apropiados, con las competencias pertinentes, producción y combinación de habilidades que se requieren para satisfacer las necesidades de salud de la población.

Se entiende por accesibilidad: la distribución equitativa de los profesionales de la salud en términos de tiempo de viaje y transporte (espacial); horario de funcionamiento y presencia de fuerza de trabajo correspondiente (temporal); atributos de infraestructura (físicos, por ejemplo, edificios aptos para discapacitados); mecanismos de referencia (organizativos) y recursos de costos directos e indirectos de los servicios, tanto formales como informales (financieros)^10^.

Para estructurar la recolección de datos se utilizó la herramienta Cuentas Nacionales del Personal de Salud (CNPS), desarrollada por la Organización Mundial de la Salud (OMS) versión en portugués (2020)^11^. El análisis que se presenta en este artículo se centró en la disponibilidad de los profesionales, en términos de cantidad (densidad con respecto a la población nacional) y composición (género, edad, nivel de formación y proporción entre enfermeros y médicos); y la accesibilidad espacial (distribución por regiones), en la diferenciación por categorías, según la Figura 1.

**Figura 1:** Indicadores de las Cuentas Nacionales de Personal de Salud e indicadores complementarios seleccionados para la recolección y el análisis de la información

Elemento del marco conceptual AAAQ*	Área del indicador	Nombre del indicador	Descripción utilizada
Disponibilidad	Número de trabajadores de la salud activos	Densidad de profesionales de la salud.	Numerador: Número de profesionales de la salud, definido como número de personas. Denominador: Población total.
Disponibilidad	Distribución	Distribución de profesionales por franja etaria.	Numerador: Número de profesionales de la salud activos en una franja etaria específica. Denominador: Número total de profesionales de la salud activos, definido como número de personas.
Accesibilidad	Número de trabajadores de la salud activos	Densidad de profesionales a nivel subnacional - regional.	Numerador: Número de profesionales de la salud activos en unidades administrativas subnacionales, definido como número de personas. Denominador: Población total a nivel subnacional.
Disponibilidad	Complementario	Razón entre profesionales de enfermería y médicos: nacional y regional y por categoría.	Numerador: Número total de enfermeros/profesionales de enfermería habilitados para ejercer. Denominador: Número total de médicos habilitados para ejercer^†^.

*La estructura AAAQ es una secuencia de cuatro dimensiones críticas para analizar: Disponibilidad; Accesibilidad; Aceptabilidad y Calidad (del inglés *Availability, Accessibility, Acceptability and Quality*); †Según la Organización para la Cooperación y el Desarrollo Económico (OCDE)(12), se considera profesional habilitados para la práctica al que cuenta con requisitos de formación y calificación y licencia (registro/habilitación) para ejercer la profesión. El concepto de profesional activo se refiere al que cuenta con formación y cualificación como requisitos previos para prestar servicios a pacientes y comunidades, de gestión o docencia. El profesional en ejercicio es el que brinda servicios directos a pacientes y comunidades

### Recolección y análisis de datos

Los datos fueron identificados a través de una combinación de bases de datos disponibles en sitios *web* institucionales: a) Plataforma Red Interinstitucional de Información para la Salud (RIPSA)^13^, red compuesta por instituciones gubernamentales y no gubernamentales^14^: número de profesionales aptos para trabajar (auxiliares, técnicos y enfermeros) por sexo y por estado en los años 1990, 1995 y 2007 y cantidad de profesionales (auxiliares, técnicos y enfermeros) inscriptos por estado en los años 1990 a 2008 y 2010; b) Consejo Federal de Enfermería (Cofen)^9^, organismo responsable de regular las profesiones relacionadas con la enfermería en Brasil, que incorpora datos del Consejo Regional de Enfermería (Coren) a través del sistema Cofen/Coren: número de profesionales aptos para trabajar por estado, categoría, edad y sexo de los profesionales registrados; c) Cuentas Nacionales de Personal de Salud (CNPS)^15^, desarrollada por la Organización Mundial de la Salud (OMS), que se basa en el marco conceptual del mercado laboral de salud^11^: datos sobre sexo, edad de los profesionales de enfermería para los años 2014, 2017, 2019, que pueden variar entre habilitados para ejercer y en ejercicio; y d) Instituto Brasileño de Geografía y Estadística (IBGE)^16^, principal proveedor de datos e información del país, que satisfacen las necesidades de diferentes segmentos de la sociedad civil y de las agencias gubernamentales^17^: los datos relativos a la población brasileña, por unidad federativa y por región, fueron obtenidos a través de estimaciones poblacionales del IBGE publicadas en el Diario Oficial de la República.

Aunque la mayoría de los indicadores propuestos consideren solamente a los profesionales activos, el nivel de actividad de los profesionales utilizados en los análisis varió en función de la información disponible en las bases de datos. También se recopiló información disponible en informes publicados, tales como: a) Informe Integrado de Gestión del Consejo Federal de Enfermería de 2019^18^: presenta el número y género de los profesionales (enfermeros y auxiliares y técnicos en enfermería) por estado para el año 2019; b) Informe de la Comisión de *Business Intelligence* Producto 2^19^: muestra datos de los profesionales de enfermería registrados en los Consejos Regionales de Enfermería (Coren) en los estados por categoría profesional, sexo y franja etaria en 2010; c) Informe de la Comisión de *Business Intelligence* ^20^: con datos de los profesionales de enfermería registrados en los Coren en los estados por categoría profesional, sexo y franja etaria en 2011, y d) Perfil de la Enfermería en Brasil (Informe Final)^21^, producto del proyecto de investigación cuyo objetivo central fue determinar el perfil del equipo de enfermería en Brasil. En el respectivo documento se identificaron los profesionales de enfermería por franja etaria en 2013.

La recolección de datos fue realizada entre diciembre de 2022 y abril de 2023 por dos investigadores simultáneamente. Si bien los autores siguieron el mismo método de búsqueda de datos para cada una de las bases de datos mencionada anteriormente, se identificó que faltaba información, lo que imposibilitó la recolección secuencial de todos los años, en todas las bases de datos utilizadas.

Los datos obtenidos se organizaron en la aplicación Excel y se calcularon los indicadores descritos en la Figura 1, considerando, además del incremento porcentual (tasa de crecimiento), el promedio de profesionales por sexo, por categoría (enfermeros, técnicos y auxiliares de enfermería) y por edad.

En lo que respecta al Comité de Ética, a pesar de ser un estudio descriptivo, este artículo forma parte de un estudio mayor, que fue presentado y aprobado por el Comité de Ética de la Escuela de Enfermería de Ribeirão Preto (CAAE: 43184621.0.0000.5393 y Número de Dictamen: 4.859.633).

## Resultados

### Disponibilidad de la fuerza de trabajo

El número total de registros profesionales de enfermería en el sistema del Consejo Federal de Enfermería (Cofen) y del Consejo Regional de Enfermería (Coren) era de 2.767.741 en diciembre de 2022^9^. Al analizar la composición, se pudo identificar que está compuesta en su mayoría por profesionales de enfermería de nivel medio y técnicos, 75,47% (en total 2.088.700 técnicos y auxiliares de enfermería) y por una minoría del 24,53% con educación superior (679.041 enfermeros). Con respecto a la densidad de profesionales de enfermería por cada mil habitantes en Brasil, vimos un aumento significativo entre 1990 y 2022, pasó de 0,96 a 12,97, respectivamente. Si bien este aumento es constante y se observa a lo largo de los años, es muy significativo en el período de 2005 a 2010, cuando la densidad de profesionales en el país prácticamente se duplicó en cinco años, al pasar de 3,82 a 7,58, respectivamente. Los responsables de ese aumento fueron los profesionales de nivel medio, los técnicos en enfermería pasaron de 0,94 a 3,24 y los auxiliares de 2,33 a 2,80 en ese período. Otro momento que hay que observar es el período de 2019 a 2022, que abarca la pandemia, en el que el crecimiento de la densidad de profesionales en tres años fue casi del 22% (de 10,64 a 12,97), mientras que, en el período anterior, el crecimiento fue aproximadamente del 14% (de 9,35 a 10,64) (Figura 2).

**Figura 2:** Evolución del número y de la densidad de profesionales de enfermería por cada mil habitantes. Brasil, 1990-2022

Fuente: Número de registros de profesionales de enfermería por categoría: Consejo Federal de Enfermería (Cofen)^9^ y Red Interinstitucional de Información para la Salud (RIPSA)^13^. Datos de la población obtenidos por el Instituto Brasileño de Geografía y Estadística (IBGE)^16^

En cuanto a la caracterización de los profesionales, al analizar la distribución de los profesionales de enfermería (sin distinción de categorías) por sexo, se observa que, a pesar de que hubo un ligero aumento en el número de profesionales masculinos, las mujeres seguían predominando en las dos últimas décadas, de 1990 a 2019, y representaron el 88,00% en 2019, como se indica en la Figura 3, inciso (a). Un análisis más detallado de las categorías profesionales en el período de 1990 a 2011 revela que hubo un aumento ligeramente más pronunciado del contingente masculino en los profesionales de nivel superior, que pasó del 6,42% en 1990 al 11,86% en 2011 (Figura 3, subítem b).

**Figura 3:** Caracterización de la fuerza de trabajo de enfermería por sexo, total y por categoría. Brasil, 1990-2019

Fuente: Número de registros profesionales de enfermería, 2011: Consejo Federal de Enfermería, Cofen^20^; 1990 a 2010: Red Interinstitucional de Información para la Salud (RIPSA)^13^ y; 2014 a 2019: Cuentas Nacionales del Personal de Salud (CNPS)^15^; Número de registros de profesionales de enfermería por categoría y por sexo: Consejo Federal de Enfermería (Cofen)^19^ ^)-(^ ^20^ y Red Interinstitucional de Información para la Salud (RIPSA)^13^

Durante el período de 2010 a 2019, al analizar la distribución por edades de los profesionales de enfermería, se identificó que, a pesar de que los datos provenían de diferentes fuentes, predominaron los individuos entre 36 y 55 años. Sin embargo, en 2017 y 2019 se produjo una disminución del porcentaje de profesionales menores de 35 años. Los datos revelaron que los profesionales menores de 35 años representaban más del 35% en todos los años estudiados, y osciló entre el 35,70% y el 45,90%, mientras que los de 56 años o más representaban más del 6% del total de profesionales de enfermería, con porcentajes que van entre 6,09% y 9,81%. Esta información se obtuvo del número de registros de profesionales de enfermería por categoría en los años 2010 y 2011^9^, en 2013^21^ y, en 2014, 2017 y 2019, presente en las CNPS^15^.

Para analizar la combinación de habilidades se utilizó la densidad y la razón entre profesionales de enfermería y médicos en el país, Tablas 1 y 2. Cuando comparamos los datos de la densidad de enfermeros con la de médicos por cada 10 mil habitantes para el año 2022, la de los enfermeros fue ligeramente mayor (31,83 y 27,38 respectivamente), y alcanzó una razón nacional de 1,16 enfermeros por médico el mismo año. La mayor diferencia entre las densidades de estos profesionales se observa en las regiones Norte y Nordeste, que también presentaron la mayor proporción de enfermeros por médico del país (1,98 y 1,64 respectivamente). En el otro extremo, están las regiones Sur y Sudeste, que presentaron las menores proporciones de enfermeros por médico (0,95 y 0,99 respectivamente). Al analizar la razón entre profesionales de enfermería y médico, se identificó que la razón nacional era de 4,74, y varió entre 3,83 profesionales de enfermería por médico en la región Sur y 8,52 en la región Norte.

**Tabla 1:** Densidad de profesionales, total y por categoría, por 10.000 habitantes y razón de profesionales de enfermería, total y enfermeros, por médico. Brasil, 2022

Región	Densidad por 10 mil habitantes							Razón	
	ENF^†^	Téc Enf^‡^	Aux Enf^§^	Prof Enf^ǁ^	Médicos*	ENF^†^ y médicos	Prof Enf^ǁ^ y médicos	ENF^†^ por médico	Prof Enf^ǁ^ por médico
Norte	28.72	85.65	9.29	123.66	14.52	43.21	138.18	1.98	8.52
Nordeste	31.64	72.40	11.71	115.75	19.29	50.92	135.04	1.64	6.00
Sudeste	33.56	78.67	34.87	147.10	33.90	67.46	181.00	0.99	4.34
Sur	26.80	73.22	13.07	113.09	29.52	56.32	142.61	0.95	3.83
Centro-Oeste	35.91	78.39	7.89	122.19	31.02	66.93	143.21	1.16	3.94
Total	31.83	76.79	21.12	129.75	27.38	59.22	157.13	1.16	4.74

Fuente: Número de registros de profesionales de enfermería por categoría: Consejo Federal de Enfermería (Cofen)^9^; número de habitantes (población brasileña) em 2022: Instituto Brasileño de Geografía y Estadística (IBGE)^16^ y número de registros de profesionales médicos: Demografía Médica en Brasil 2023^22^

*Se utilizó el número de registros de profesionales médicos para poder compararlos con los datos de enfermería; ^†^ENF = Enfermeros; ^‡^Téc Enf = Técnicos en enfermería; ^§^Aux Enf = Auxiliares de enfermería; ^ǁ^Prof Enf = Profesionales de enfermería

**Tabla 2:** Número de profesionales de enfermería, total y por categoría, y tasa de crecimiento por región. Brasil, 2000 e 2022

Región	ENF*			Téc Enf^†^			Aux Enf^‡^			Prof Enf^§^		
	2000	2022	Tasa	2000	2022	Tasa	2000	2022	Tasa	2000	2022	Tasa
Norte	4.150	54.305	1209%	3.408	161.936	4652%	13.683	17.559	28%	21.241	233.800	1001%
Nordeste	17.959	182.442	916%	10.912	417.526	3726%	52.550	67.544	29%	81.421	667.512	720%
Sudeste	38.054	300.804	690%	44.627	705.131	1480%	185.621	312.521	68%	268.302	1.318.456	391%
Sur	9.263	81.487	780%	8.822	222.594	2423%	42.059	39.746	-5%	60.144	343.827	472%
Centro-Oeste	4.695	60.003	1178%	10.074	130.965	1200%	16.254	13.178	-19%	31.023	204.146	558%
Total	74.121	679.041	816%	77.843	1.638.152	2004%	310.167	450.548	45%	462.131	2.767.741	499%

Fuente: Número de profesionales de enfermería registrados por categoría en 2022, obtenido a través del Consejo Federal de Enfermería (Cofen)^9^, y en el 2000, obtenido por medio de la Red Interinstitucional de Información para la Salud (RIPSA)^13^

*ENF = Enfermeros; ^†^Téc Enf = Técnicos en enfermería; ^‡^Aux Enf = Auxiliares de enfermería; ^§^Prof Enf = Profesionales de enfermería

Al analizar la disponibilidad del grupo de profesionales en el país, como se muestra en la Tabla 1, se observó que la densidad nacional era de 59,22 enfermeros y médicos, y de 57,13 profesionales de enfermería y médicos por cada 10 mil habitantes. Además, se pudo identificar que la menor densidad del país la registraban las regiones Norte y Nordeste, tanto de profesionales con educación superior (43,21 y 50,92 enfermeros y médicos por cada 10 mil habitantes, respectivamente), como de profesionales de nivel medio/técnico (138,18 y 135,04 profesionales de enfermería y médicos por cada 10 mil habitantes).

### Accesibilidad de la fuerza de trabajo

Al analizar la distribución de los profesionales de enfermería en las diferentes regiones de Brasil, se observa que el mayor número se concentra en la región Sudeste, seguida de la región Nordeste, y se observó la misma distribución en las diferentes categorías profesionales. La región Norte se presenta como la región con menor número de enfermeros del país, sin embargo, al analizar el número de profesionales de enfermería, auxiliares y técnicos en enfermería, la región con menor representación es la Centro-Oeste (Tabla 2).

Si bien la región Sudeste tiene la mayor representación del país con respecto al número de profesionales de enfermería en sus diferentes categorías, fue la que mostró el menor crecimiento durante el período de 2000 a 2022, considerando el número total de profesionales de enfermería y enfermeros. La región Norte fue la de mayor crecimiento en relación a las diferentes categorías analizadas, con excepción de los auxiliares de enfermería. Sin embargo, cuando se observaron sólo los auxiliares de enfermería, las regiones Sur y Centro-Oeste registraron una disminución en la categoría (Tabla 2).

Por lo tanto, es importante destacar la densidad de profesionales, es decir, el número de profesionales por habitantes en un determinado lugar. En relación a este indicador, la región Sudeste, a pesar de presentar la menor tasa de crecimiento, en el período analizado en este estudio, es la región con mayor densidad de profesionales de enfermería (y número de profesionales) del país, ya que tiene la segunda mayor densidad de enfermeros y la mayor densidad de profesionales de nivel medio y técnico, especialmente de auxiliares de enfermería (34,87 en contraste con la desaceleración que presenta esta categoría). Si se analiza la densidad de enfermeros en todas las regiones del país, la región Centro-Oeste ocupa el primer lugar, aunque tenga el cuarto menor número de enfermeros.

La región Nordeste, a pesar de destacarse por el número de personal de enfermería, y ubicarse en la segunda posición en todas las categorías, tiene la segunda menor densidad de profesionales de enfermería y la menor densidad de técnicos y auxiliares juntos. El gran crecimiento de la región Norte aún no se refleja en mejores densidades profesionales, dado que presenta la segunda densidad más baja a nivel nacional. La región Sur tiene la menor densidad de profesionales de enfermería y enfermeros del país y la segunda mayor densidad de auxiliares de enfermería (Tabla 1).

## Discusión

Se determinó que la densidad de enfermería en Brasil, de aproximadamente 130 profesionales de enfermería y 32 enfermeros por cada 10.000 habitantes en 2022, es superior al promedio de los países de la Región de América Latina y el Caribe (ALC) y de la Organización para la Cooperación y el Desarrollo Económico (OCDE), que presentan poco más de 35 y 103 profesionales de enfermería por cada 10 mil habitantes, respectivamente, el mismo año (información disponible para ALC en 33 países, y OCDE para 38 países)^23^, lo que demuestra un aparente “exceso de profesionales” en Brasil. Es importante destacar que los datos globales pueden estar considerando profesionales en ejercicio y/o activos, mientras que los datos analizados de Brasil se refieren a los profesionales habilitados para la práctica, considerando el número de registros en el Sistema Cofen/Coren.

Además, en Brasil, en 2022, siete de cada diez profesionales de enfermería tenían nivel medio y/o técnico (75%), contrariamente a lo que se observa en el escenario mundial, en el que el 69% de los profesionales tenía nivel superior (considerando datos de años anteriores). En Europa, los enfermeros constituyen el 70% de la fuerza de trabajo de enfermería (por ejemplo, en el Reino Unido el 80% de los profesionales tiene educación superior)^24^. Excepto en algunos países, como Canadá y Estados Unidos (el 75,46% y 85,74% de los profesionales tiene educación superior, respectivamente)^15^ ^),(^ ^24^, la fuerza de trabajo de enfermería en el continente americano está compuesta en su mayoría por profesionales de nivel medio y/o técnicos, pero en menor proporción que en Brasil (59%)^25^.

Durante el período analizado (1990 a 2022), se identificaron dos momentos en los que hubo un aumento significativo del número de profesionales de enfermería en Brasil. El primero se produjo entre 2005 y 2010, cuando se produjo un aumento importante de la densidad de profesionales, que casi se duplicó al pasar de 3,82 a 7,58. Los técnicos en enfermería jugaron un papel fundamental en ese aumento, que pasaron de 0,94 a 3,24. Ese incremento fue impulsado por estrategias nacionales, como el Proyecto de Profesionalización de Trabajadores del Área de Enfermería (PROFAE)^26^. El segundo período de crecimiento de la densidad de profesionales de enfermería, que fue cerca del 22% entre 2019 y 2022, contradice parcialmente los resultados de una revisión integradora internacional que analizó 43 estudios (pre y post-COVID-19) e indicó que hubo un aumento significativo de la tasa de intención de abandonar la profesión después de la pandemia de COVID-19, observación que también realizó el *International Council of Nurses* (ICN). Una investigación con las Asociaciones Nacionales de Enfermería reveló que se registró un aumento del número de enfermeros que abandonan la profesión. En ese contexto, se destaca que los factores relacionados con esa intención y/o abandono son: gran carga de trabajo, recursos insuficientes, agotamiento físico y estrés^27^ ^)-(^ ^28^. Por lo tanto, cabe destacar que es imperativo examinar este escenario en Brasil, y analizar no sólo el número de profesionales involucrados en la atención directa a los pacientes y la comunidad, sino también el número de personas que optaron por abandonar esta noble profesión.

A pesar de ser una profesión joven, en la cual más de un tercio de los profesionales se encuentra en el inicio de su carrera y menos del 9% cerca de jubilarse (2019), la profesión está envejeciendo en el país (2010 a 2019). Una fuerza de trabajo de enfermería más joven puede ayudar a garantizar la sostenibilidad de la profesión de enfermería. A medida que los enfermeros de mayor edad se jubilan, es importante contar con una nueva generación que las reemplace y continúe brindando atención de alta calidad^29^. A pesar de que la situación dista mucho de la de algunos países de América, cuyas poblaciones están muy envejecidas, como Panamá y República Dominicana (donde más del 26% de los profesionales tiene 55 años o más)^15^, y países de Europa, por ejemplo, Bulgaria, Islandia y la República de Moldavia (donde aproximadamente el 40% de los profesionales tiene 55 años o más)^30^, esta información sugiere que es necesario monitorear este indicador y realizar análisis en las diferentes categorías, para comprender mejor los flujos de entrada y salida y las necesidades de los profesionales.

En cuanto a las características de la fuerza de trabajo de enfermería, predominan los profesionales del sexo femenino. En este estudio, se identificó que hubo un aumento del contingente masculino, que representaba el 12% de los profesionales habilitados para ejercer en 2019, el aumento fue ligeramente mayor en la categoría enfermeros (del 6% al 12% 1990 - 2011). Los datos sobre los profesionales de enfermería en ejercicio indican que el porcentaje era del 13,7% en 2021 para Brasil^16^. Estos valores son cercanos a los identificados en otros países de América, con un 13% en 2019^16^, entre los que se destaca Argentina, donde aproximadamente el 20% de los profesionales de enfermería eran del sexo masculino en 2020^15^ ^),(^ ^25^.

Aunque los profesionales de la salud del sexo femenino sean responsables de brindar la mayor parte de la atención en todos los niveles y entornos, y representan alrededor del 67% de los trabajadores asalariados en el sector de la salud, se encuentran con barreras en el trabajo, tales como prejuicios, discriminación, acoso sexual y violencia, además de los riesgos desproporcionados que surgieron durante la pandemia de COVID-19^31^. Los profesionales del sexo masculino no enfrentan esas barreras. Es necesario incorporar el género y la edad en las estrategias de la FTS para garantizar que se adopten enfoques sensibles y basados en evidencia para la planificación y gestión de los profesionales. La falta de datos precisos sobre la composición por género y edad en varios países impide mejorar las condiciones de trabajo y las estructuras profesionales para apoyar la participación y el avance de las mujeres en la FTS, así como también valorar las contribuciones de las mujeres y de los profesionales en los sistemas de atención médica^32^. 

Al estimar la combinación de habilidades de la FTS mediante la comparación entre la razón de profesionales, se obtiene información sobre la posibilidad de mejorar la eficiencia y también la proporción de enfermeros por médico como potencial indicador de la calidad de la atención al usuario^2^ ^),(^ ^33^. Brasil presentó una razón nacional en 2022 de 1,16 enfermeros y dos de sus regiones, la Región Sur y Sudeste, presentaron razón de enfermeros por médico inferiores a 1 (0,95 y 0,99 respectivamente). Se identificó que esos datos eran inferiores al promedio de los países de ALC y de la OCDE, que corresponden a 1,9 y 2,7 enfermeros por cada médico, respectivamente, para el mismo año (información disponible para ALC de 32)^23^. Varios países de la región tienen tasas superiores a este valor, entre ellos Dominica, la más alta de la región, con 5,5 enfermeros por médico. Por otro lado, se destaca la razón de 0,6 enfermeros por médico en Colombia, en el extremo más bajo de la región de ALC.

Este estudio identificó una proporción nacional en Brasil de profesionales de enfermería por médico de 4,74 en 2022. Si se considera el promedio de la OCDE y de ALC, esta proporción es relativamente mayor en Brasil. Además, es importante recalcar que la fuerza de trabajo de enfermería tiene características diferentes (está compuesta principalmente por profesionales de nivel medio y técnicos). Cabe destacar que, el documento de la OCDE presenta datos sobre los profesionales activos en Brasil en 2019 (3,4 profesionales de enfermería) e identifica que el valor es superior al de estos grupos de países (el promedio de la OCDE es de 2,6 y el de ALC es de 1,6)^34^. Por lo tanto, es fundamental repensar la combinación de habilidades profesionales de la salud en el contexto de un envejecimiento de la población y un aumento de la carga de enfermedades crónicas en el país.

Teniendo en cuenta la accesibilidad a la fuerza de trabajo, las estimaciones de densidad informan la disponibilidad *per cápita* de profesionales de la salud, del mismo modo que la variación en la densidad entre regiones geográficas proporciona información sobre la distribución equitativa de los profesionales de la salud en las regiones^2^. El documento de base para la Estrategia Mundial de Recursos Humanos para la Salud: personal sanitario 2030 de la OMS estimó que el “límite mínimo” necesario para brindar cobertura sanitaria universal a la población es de 44, profesionales de la medicina, enfermería y partería por cada 10.000 habitantes^35^ ^)-(^ ^36^. Al analizar la disponibilidad del conjunto de profesionales en el país, este estudio observó que la densidad nacional era de 59,22 enfermeros y médicos por cada 10 mil habitantes, que si se incluyen los profesionales de nivel medio y técnicos la densidad es de 157,13 profesionales de enfermería y médicos, es decir, ambas densidades nacionales alcanzan el “límite mínimo”. Al considerar sólo a los profesionales con formación superior, la región Norte tuvo una densidad inferior a ese límite establecido (43,21 enfermeros y médicos por cada 10 mil habitantes). Sin embargo, aunque ese límite sea una métrica valiosa para realizar comparaciones a nivel internacional y seguimiento, no es aconsejable utilizarlo directamente para establecer objetivos nacionales. Este enfoque ignora las particularidades del país, al igual que las condiciones de salud, las estructuras y la presencia de otros grupos ocupacionales diferentes a las tres profesiones existentes en el país. Por lo tanto, es fundamental desarrollar metas adaptadas al contexto nacional, considerando las prioridades políticas para la disponibilidad y composición de la Fuerza de Trabajo en Salud (FTS)^2^.

En las últimas dos décadas, hubo un movimiento para ampliar la formación de la Enfermería en Brasil, con la creación de escuelas en todas las regiones. Este movimiento surgió de la democratización del acceso a la educación superior e hizo que hubiera una mayor disponibilidad de enfermeros en el mercado^37^. A pesar de esa expansión, las Escuelas de Enfermería se concentran en los estados con mayor densidad poblacional y concentración de ingresos del país, sigue la distribución del Producto Interno Bruto (PIB) y se perpetúan las desigualdades económicas y sociales en las regiones brasileñas^37^.

Los datos identificados en este estudio, para el año 2022, indican que sólo la región Sudeste contaba con casi el 50% de los profesionales de enfermería del país. Además, esta región también presentó mayor número de profesionales en años anteriores, según un estudio que analizó información de enfermeros en diferentes años de la década de 2010^38^. Las zonas remotas pueden enfrentar escasez de profesionales cualificados, lo que prolonga las disparidades regionales. Mientras que, las zonas centrales y ricas pueden tener una menor oferta de profesionales cualificados y una menor empleabilidad^39^.

Un estudio buscó estimar el número de enfermeros empleados que deberían ser redistribuidos para que haya una distribución geográfica más adecuada de estos profesionales en Brasil, e identificó que la región Sudeste debería redistribuir el 8,8% de sus enfermeros entre las regiones brasileñas, lo que representa alrededor de 6 mil puestos de trabajo de enfermería que deberían ser redistribuidos para lograr la igualdad^40^. Además, un análisis sobre la cobertura poblacional de enfermeros, en los estados de la región Norte y Nordeste, demostró que la cobertura poblacional era inferior a 5 enfermeros por cada 10 mil habitantes, con valores superiores a 10 enfermeros por cada 10 mil habitantes^38^.

Las limitaciones de este estudio se deben a las características de cada una de las bases de datos consultada, debido a la fragmentación de la información y al uso de diferentes metodologías en la recolección de datos, así como al nivel de desempeño profesional. Cabe destacar que es necesario estandarizar la recolección y difusión de datos de los profesionales de la salud.

Es fundamental realizar el seguimiento y la evaluación continuos de la fuerza de trabajo de enfermería. Por ende, considerando la desigualdad de la distribución del número de profesionales de enfermería en los estados brasileños, es importante que los poderes públicos sean los responsables de este proceso y dicten cursos de enfermería adaptados a las realidades locales y a las necesidades globales de formación, en los territorios que carecen de enfermeros, especialmente en el interior de Brasil^41^.

En definitiva, este estudio juega un papel importante en el avance del conocimiento científico, al proporcionar un análisis detallado de la disponibilidad y accesibilidad de la fuerza de trabajo de enfermería en Brasil. Al emplear conjuntamente métodos descriptivos y transversales e indicadores cuidadosamente seleccionados, los investigadores pudieron obtener una visión general completa de la situación. Los resultados destacan que hubo cambios en el tiempo, y revelan un notable aumento en el número de profesionales de enfermería, especialmente de nivel medio y técnico, entre 2005 y 2010. Además, se identificó que la distribución de los profesionales en las diferentes regiones de Brasil era desigual, y que la región Sudeste era la que tenía la mayor cantidad. El análisis también destacó que persisten ciertos desafíos, como el predominio del sexo femenino en todas las categorías y la necesidad de implementar políticas públicas más sólidas para diversificar y redistribuir los profesionales en todo Brasil. Estos hallazgos no solo brindan información para la toma de decisiones futuras en las áreas de atención médica y educación, sino que también brindan información valiosa para formular estrategias que mejoren la calidad de la atención y promuevan la equidad en el acceso a los servicios de salud en todo el país.

Por lo tanto, se destaca lo importante que es implementar políticas públicas que consideren las estadísticas para la planificación y ejecución de políticas y estrategias, con el fin de reconfigurar la distribución de los profesionales para promover una asignación más equitativa en todas las regiones del territorio brasileño. Hay que destacar el esfuerzo realizado con el Programa de Valorización de Profesionales de Atención Primaria (PROVAB), cuyo objetivo es proveer equipos de Atención Primaria y Estrategia Salud de la Familia con enfermeros, odontólogos y médicos a las zonas alejadas y más vulnerables.

Por último, es muy importante que se realicen nuevas investigaciones y que se implementen políticas que analicen en profundidad la eficacia de integrar a los profesionales en sus funciones y lugares de trabajo. Esto es crucial para comprender la creciente demanda de profesionales de enfermería, una necesidad que parece intensificarse cada año a pesar del aumento del número de estos profesionales.

## Conclusión

A pesar de la gran cantidad de personal de enfermería, existe una disparidad en la distribución en todo el país. Además, se puede observar que no solo hay menor número de enfermeros, sino que presentaron un crecimiento mucho menos pronunciado que los técnicos en enfermería en algunas regiones de Brasil, por lo que el crecimiento de los técnicos en enfermería superó al de los enfermeros, lo que indica que las políticas de formación técnica son más intensivas que las de educación superior.
